# When the antidote is the poison: Investigating the relationship between people’s social media usage and loneliness when face-to-face communication is restricted

**DOI:** 10.1371/journal.pone.0296423

**Published:** 2024-02-09

**Authors:** David Jütte, Thorsten Hennig-Thurau, Gerrit Cziehso, Henrik Sattler

**Affiliations:** 1 Marketing Center Münster, University of Münster, Münster, Germany; 2 Marketing & Branding, University of Hamburg, Hamburg, Germany; The Hong Kong Polytechnic University, HONG KONG

## Abstract

When governments mandated lockdowns to limit the spread of the coronavirus, the resulting reduction of face-to-face communication threatened many people’s psychological well-being by fostering feelings of loneliness. Given social media’s eponymous social nature, we study the relationship between people’s social media usage and their loneliness during these times of physical social restrictions. We contrast literature highlighting the social value of social media with a competing logic based on the “internet paradox,” according to which increased social media usage may paradoxically be associated with increasing, not decreasing, levels of loneliness. As the extant literature provides opposing correlational insights into the general relationship of social media usage and loneliness, we offer competing hypotheses and offer novel longitudinal insights into the phenomenon of interest. In the empirical context of Germany’s initial lockdown, our research uses survey panel data from February 2020 (before the lockdown) and April 2020 (during the lockdown) to contribute longitudinal evidence to the matter. We find that more usage of social media in the studied lockdown setting is indeed associated with *more*, not less loneliness. Thus, our results suggest a “social media paradox” when physical social restrictions are mandated and caution social media users and policy makers to not consider social media as a valuable alternative for social interaction. A post-hoc analysis suggests that more communication via richer digital media which are available during physical lockdowns (e.g., video chats) softens the “social media paradox”. Conclusively, this research provides deeper insights into the social value of social interactions via digital media during lockdowns and contributes novel insights into the relationship between social media and loneliness during such times when physical social interaction is heavily restricted.

## Introduction

As one of the most prominent and damaging global crises in recent decades, the COVID-19 pandemic prompted unprecedented developments. Over its course, politicians in many countries ordered so-called “lockdowns,” in which governmental authorities required people to restrict their physical movement and reduce their face-to-face contact with others in an effort to limit the spread of the virus [1]. Although these restrictions were effective measures in reducing the individual probability of contagion [2, 3], the decreed physical distancing has also challenged people’s psychological well-being as it fostered social isolation [4–7]. For many people, this social isolation was associated with an unmet need for social interaction which often increased loneliness during this time of physical social distancing [8, 9].

Whereas face-to-face interaction was restricted by lockdown measures, channels for digital interaction generally remained accessible, allowing people to shift their otherwise restricted social interactions to the digital realm. Consequently, people’s usage of social media, as one of the most popular channels for social interaction online, substantially increased during this period [10]. As social media is able to connect people and facilitate social relationships [11–13], its increased usage had the potential to support people in dealing with the extraordinary isolation during the initial COVID-19 lockdowns. Thus, people’s increased social media usage may have helped them to compensate for the loss of face-to-face interaction by addressing the need for social connectedness online, preventing people from experiencing a substantial increase of loneliness.

However, some other prior research suggests that an increase in social interaction online may paradoxically intensify people’s feelings of loneliness, not reduce them. Kraut et al. [14] coined this phenomenon the “internet paradox”, originally arguing that the usage of asynchronous applications like e-mails and online chats fosters forms of social interaction, which are rather ineffectual with regards to social connectedness as well as overall psychological well-being and which displace face-to-face interactions, which are substantially more beneficial in these regards [14]. Follow-up research on this “internet paradox” has applied this displacement logic to synchronous online interaction channels as well [15]. This provides tentative support that such a paradoxical relationship may also exist between social media and loneliness.

Overall, it is unclear whether extending the use of social media in a lockdown reduces users’ loneliness (by compensating otherwise lost face-to-face social interactions), or adds to their loneliness, thus providing a specification of the “internet paradox” in terms of a “social media paradox”. This research is situated in the context of psychological well-being as an essential topic for the social sciences [16–18]. Specifically, we focus on people’s loneliness, which has been argued to pose a risk to longevity similar to “smoking up to 15 cigarettes a day” [19] and, because of its widespread nature, has been labeled a epidemic in its own right: the epidemic of loneliness [20]. The question we want to answer with this research is: Are changes in social media usage during a government-ordered lockdown when face-to-face social interactions are restricted related to people’s loneliness, and is the relationship of a positive or negative kind?

Empirically, we use panel data we collected before and during Germany’s initial COVID-19 lockdown in the spring of 2020. This longitudinal dataset allows us to examine the relationship between the change in people’s social media usage and the loneliness they experience during the considered lockdown, as well as the relationship between people’s prior baseline social media usage and their respective loneliness. Considering the pre-lockdown usage of social media offers insights that should be less prone to reverse causal explanation approaches, as the amount of prior usage is not explained by people’s subsequently experienced level of loneliness during the time lagged event of an unanticipated lockdown. Although our statistical findings are correlational, we draw on the combined insights of our main analysis, our post-hoc analysis, and the existing related literature to argue for a possible directional relationship between social media usage and loneliness in the context of Germany’s initial lockdown.

## Theoretical development and competing hypotheses

Current research suggests that the restrictions in terms of face-to-face interaction induced by the initial COVID-19 lockdowns in 2020 were widely associated with an increase in people’s feelings of loneliness [8, 9]. However, such lockdown effects on loneliness appeared to vary across individuals [21, 22], and we assume that a consumer’s change in the use of social media as a result of lockdown restrictions for face-to-face interactions contributed to such user-level differences. The direction of this contribution is unclear though, as opposing arguments can be made based on different streams of media research.

On the one hand, the usage of social media during a lockdown gives people the opportunity to socialize online, which should help people to compensate for their experienced loss of face-to-face interactions resulting from the mandated restrictions of physical social interactions. In line with this logic, the use of social media for social motives has been generally associated with higher levels of psychological well-being and lower levels of loneliness in a number of academic studies set in non-lockdown settings [23–25]. These studies suggest that an additional usage of social media increases people’s social connectedness and reduces their loneliness. Relatedly, research has highlighted that this supposedly social nature is not only the eponym of social media, but also key driver behind its massive adoption by more than 4.5 billion people [26, 27].

Research in settings of limited face-to-face interaction, such as of community-dwelling elderlies, reports comparable effects of social media usage [28, 29]. Again, social media usage is found to increase social connectedness, pointing at the potential of social media usage to compensate for the limitations of constellations where only low levels of face-to-face interaction are possible. Consistent with this compensation logic, extant studies have linked the use of social media in a lockdown with lower levels of loneliness. Specifically, Cauberghe et al. [30] harvested cross-sectional survey data during Belgium’s initial COVID-19 lockdown in 2020, finding that during this period social media usage was associated with lower levels of loneliness in adolescents. Similarly, Bonsaksen et al. [31], again based on a cross-sectional study, report similar effects for lockdowns in multiple countries among adults of 40 years and above. The argument of social media serving as compensation for the lack of face-to-face interactions in a lockdown, supported by these tentative empirical results, leads us to offer our first hypothesis:

*Hypothesis 1 (H1)*: During a lockdown when face-to-face interaction among users is restricted, an increase in social media usage will be associated with a decrease in people’s loneliness.

On the other hand, research building on the “internet paradox” has compiled empirical support for undesirable effects of the usage of digital media on loneliness [15]. This stream of research attributes such loneliness-enhancing effects to a displacement of socially beneficial interactions by digital media usage [15]. Proponents of the “internet paradox” argue that digital media usage is ineffectual in providing social benefits for its users, and instead is crowding out more beneficial social interactions on other interaction channels, thus increasing a consumer’s feelings of loneliness.

This proposed ineffectiveness of digital media usage, which in some studies explicitly includes social media [32], has primarily been linked to two characteristics. First, digital media, in forums, bulletin boards, or internet groups, has been argued to foster social interaction with rather distant others by providing easy access to large social networks via low-effort interactions [15, 32]. Second, digital media has been said to have a rather limited ability to make people feel close to their interaction partners and to create a feeling of a shared experience. Specifically, social media has been argued to provide low social presence [33, 34] as well as a coinciding poor level of media richness [35, 36].

In contrast, other digital media such as videoconferencing software enable higher levels of social presence, are considered richer media, and also tend to be more frequently used for social interaction with closer ties in non-occupational social interactions [37, 38]. Accordingly, the use of these digital channels should provide stronger social benefits and hence be better suited to reduce feelings of loneliness than the use of social media. Applying this logic to a lockdown suggests that people should experience higher levels of loneliness when they turn to social media, as doing so implies a substitution of the remaining face-to-face communication as well as richer digital channels that would provide more social benefits (e.g., video chats).

Initial empirical support for this substitution logic comes from Li et al. [39], whose cross-sectional study with retrospective self-assessment data is situated in China’s COVID-19 lockdown in April 2020. The authors find social media usage to be associated with *increased* loneliness of Chinese citizens. Nguyen et al. [40] find similar results for cross-sectional data they collected during COVID-19 restrictions in the U.S. in 2020, but also point at their exclusiveness for social media, as their analyses for richer digital media with high levels of social presence do not produce undesirable effects on loneliness.

Notably, both studies only use data that capture during-lockdown behavior. The lack of a pre-lockdown baseline prevents them from distinguishing between (a) the *general*, i.e., not lockdown-specific, relationship between social media and loneliness and (b) the *specific* relationship between the two in the particular lockdown setting considered here, a context in which people’s physical social interaction was highly restricted. Nonetheless, these preliminary empirical results indicate that higher levels of social media were associated with higher levels of loneliness during the lockdowns considered. Thus, these findings support the idea that the usage of social media may crowd out richer digital means of social interaction, even in a lockdown setting, suggesting that a “social media paradox” may be at play during such times.

As it is unclear whether such substitution effects of social media are stronger, weaker, or of equal size than the compensation effects of social media postulated in *H1*, we offer a competing hypothesis to our original hypothesis, which assumes these substitution effects to dominate:

*Hypothesis 1A (H1A)*: During a lockdown when face-to-face interaction among users is restricted, an increase in social media usage will be associated with an increase in people’s loneliness.

## Material and methods

### Data collection

To shed light on the contradictory nature of initial cross-sectional results and to test our competing hypotheses, we leverage longitudinal data that focuses on the within-person effects of a consumer’s changed social media usage during a lockdown.

Specifically, we use the first nationwide lockdown in Germany as the contextual setting for our research. The lockdown began on March 22, 2020; the mandated restrictions included closures of educational institutions, restaurants, hotels, and stores; cancellations of cultural events; bans on meetings with people outside the household except one other person, whether in public or private; a minimum distance of 1.5 meters between persons; and movement reduced to a minimum [41]. Correspondingly, the German population initially reduced their movement by about 65 percent, relative to the pre-lockdown level; over the whole lockdown period, mobility declined by 51 percent [42]. The first easing of restrictions was introduced on April 20, 2020 [1, 43].

The data for this research come predominantly from two surveys of the same respondents. The first round of data collection was conducted February 19–29, 2020, and the second one about seven weeks later (April 8–15, 2020). The average time between individual survey completion was 48 days (Time_Min_: 39.5; Time_Max_: 54.2; Time_Mean_: 48.4, SD: 1.9). Because the time difference between the two surveys is only six–eight weeks, we assume that we can attribute changes in the respondents’ loneliness to the COVID-19 lockdown.

**Fig 1:**
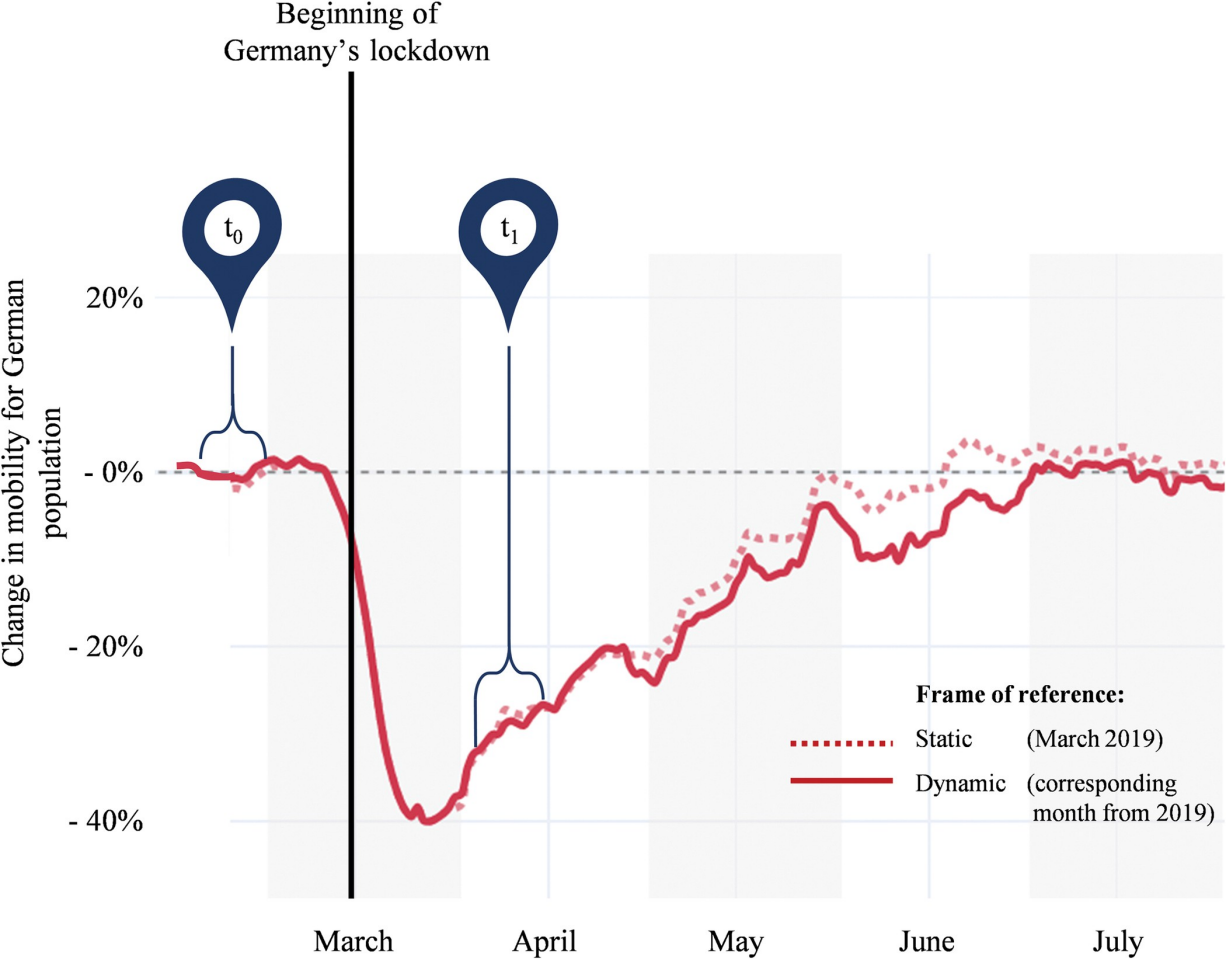
German mobility during the two survey periods. Note: The data used in Fig 1 is based on Apple’s mobility trends reported in 2020 [42].

Fig 1 illustrates the timing of the data collection and corresponding mobility levels among the German population. The first (i.e., pre-lockdown, or T1) survey, which provides baseline data for this research, took place before government pronouncements or developments in other countries hinted at an impending national lockdown [44], an unprecedented political intervention in the history of the Federal Republic of Germany. Individual mobility thus was “normal” and equivalent to the previous year at that point in time [45]. When we sent the second (i.e., during lockdown, or T2) survey, nationwide behavioral restrictions had been in place for over two weeks and applied to all participants. Mobility rates were very low, while people had gained experience with the impact of the far-reaching mandates for reducing face-to-face interactions.

The two surveys that constitute the basis for our main analysis are part of a representative nationwide panel we created in 2018 (i.e., initial survey or T0) for a longitudinal investigation of media usage in Germany, which consists of a total of four surveys (in addition to those in February and April 2020, data collection as part of the panel took place in September 2018 and in May 2019). The field work for all surveys was carried out in collaboration with the global market research company Harris Interactive AG (part of ITWP). Harris Interactive set up a Germany-wide representative panel with respect to gender, age, and education, with a target size of 2,000 participants. They were responsible of acquiring the sample, distributing the questionnaire, conducting initial quality checks, securing recontact approval from all participants and their compensation. Harris Interactive rewarded every participant who successfully completed a survey with e-points that are redeemable for items on several online platforms (e.g., Amazon). This incentive roughly equals a monetary value of 2€ per survey.

**Fig 2:**
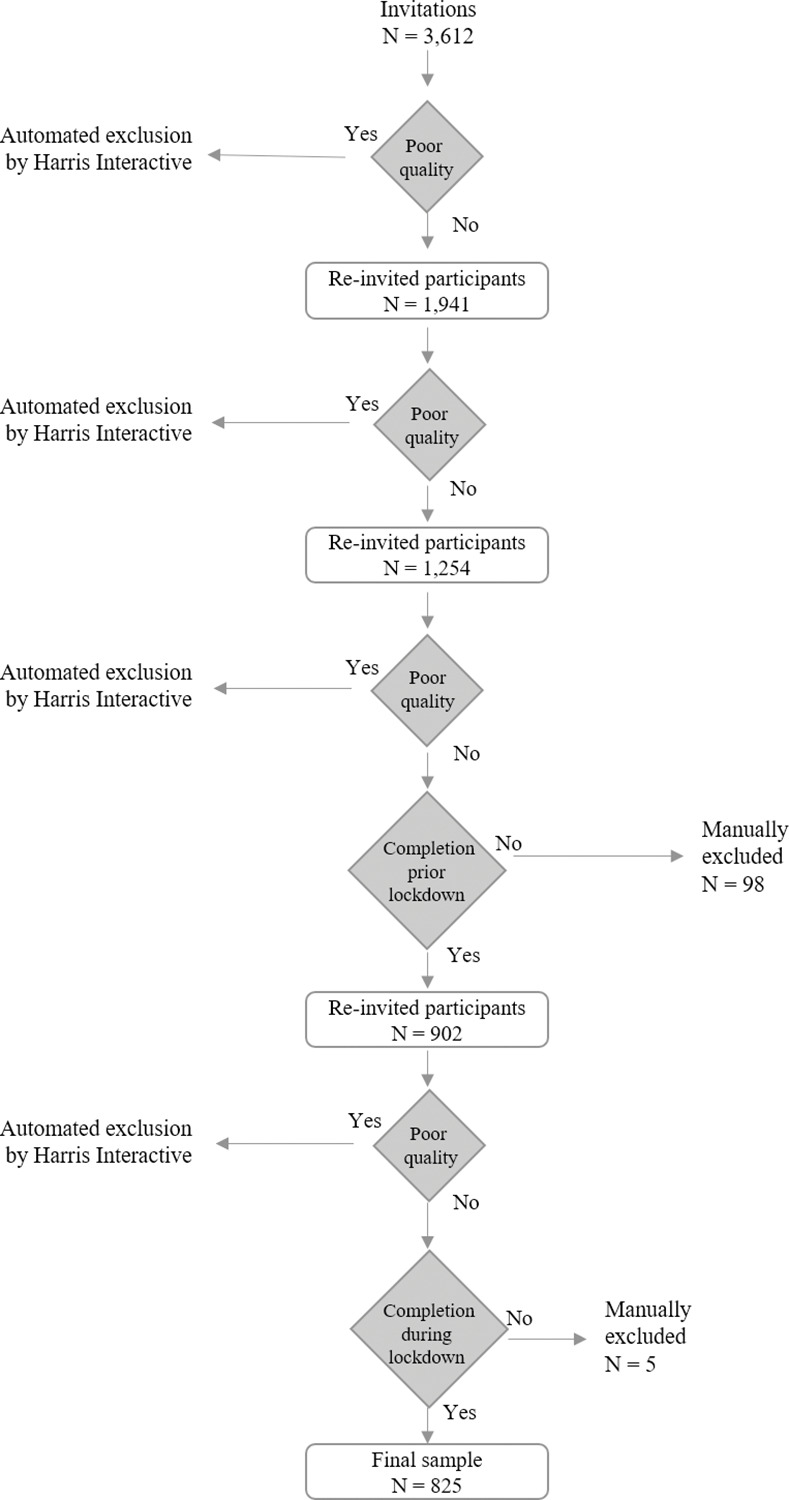
Sample acquisition and development over time.

Initially, 3,612 participants were invited to participate in the first data collection in 2018. We received a usable sample of 1,941 completed responses (dropout rate of 46%), after removing systematic response patterns (e.g., same value for each question) and implausibly fast click-through rates (faster than 9 min.). These 1,941 respondents were then re-invited to partake in the subsequent survey. This procedure was carried out analogously for the additional two surveys in terms of incentives, quality checks, and invitations. Fig 2 grants an overview of the overall panel recruitment and detailed sample cleaning process.

In addition, we further restricted our dataset to responses prior to March 1^st^ (for the pre-lockdown survey) and prior to April 15^th^ (for the lockdown survey), respectively. Since leading German politicians publicly denied the possibility of a lockdown one week prior to its introduction in March [43, 44], we assume that our respondents did not anticipate the measures that were later introduced at the time of our pre-lockdown survey. Similarly, as the loosening of lockdown measures was leaked to the public on April 15^th^ [46], we only included responses that were completed prior to that date in the lockdown survey to avoid any potential bias because of respondents’ anticipation of a loosening of lockdown restrictions. Specifically, we excluded 98 respondents who completed their answers prior to March 1^st^ as well as 5 respondents who submitted their responses after April 15^th^. Furthermore, 72 people who had completed the pre-lockdown survey dropped out in the second (lockdown) survey (dropout rate = 8%). Hence, our final sample used for the subsequent analyses of changes between the pre-lockdown survey and the lockdown survey consists of 825 responses.

### Measurement

The constructs used for the main analysis have been predominantly collected in the pre-lockdown survey (February 2020) and the lockdown survey (April 2020). Those include questions about loneliness (the dependent variable in this research), social media-related questions (the key independent variable), and several variables which we use as controls in our analysis, namely usage of other media than social media,

Exceptions are demographics (i.e., age, gender, education; measured pre-lockdown only) as well as personality variables (such as the “Big 5,” measured during the lockdown only) which we assume to be stable over the short time frame between the two rounds of data collection; these variables guided the representativeness of this panel and served as controls in our analysis. Also, the pre-lockdown information on non-occupational communication via video chats and face-to-face communication mark exceptions, as we measured them in the lockdown survey, but took the pre-lockdown information on these variables from the initial round of data collection in T0 (i.e., September 2018). They were not part of the February 2020 survey, but we assume such communication to be relatively stable across respondents prior to the lockdown. The lockdown survey also included a number of COVID-19-specific questions which allow us to control for potential changes in a respondent’s loneliness caused by the pandemic affected his/her personal and work life. Both surveys also included a number of additional information which was not used for this research, namely life satisfaction, cognitive and emotional empathy, aggressiveness, affect with regards to Germany’s political situation, respondents’ social ties and their newsfeed; the lockdown survey also contained questions regarding the respondent’s factual knowledge about the virus and conspiracy beliefs about it.

Regarding the individual scales we used, we measured people’s loneliness with three items from the revised UCLA-Loneliness scale (7-point scales; 1 = never, 7 = very frequently; How often do you feel. . .”. Item 1:..alone?, Item 2:..lonely?, Item3:..left out?) [47]. We measure change in social media usage as the change in hours spent on social media during the lockdown minus the hours of pre-lockdown social media usage. For both periods we asked the respondents to approximate their average total weekday usage of social media in hours (“How many hours do you spend on an average weekday using social media (e.g., Facebook, Twitter, Instagram etc.)?”). Additionally, we include the pre-lockdown social media usage (“baseline”) and pre-lockdown loneliness measures as a proxy for the respondent’s baseline to control for general social media intensity effects and avoid biasing the lockdown specific effect.

To address potential confounding effects of general media consumption, we also captured people’s total usage of other media (i.e., sum of self-reported hours spent with online and offline newspapers and websites, radio, television, blogs, and podcasts). As with social media usage, we use both the baseline (pre-lockdown) usage of other media and the change in media usage between lockdown and pre-lockdown (i.e., “change in other media usage”). Moreover, we include the baseline and the change in intensity of non-occupational communication via video chats (on a 5-point intensity scale, 1 = never, 5 = multiple times a day) and face-to-face communication (in hours) to control for differing levels of respondents’ communication during the considered lockdown.

For measuring how the COVID-19 pandemic affected the individual respondents’ personal and work lives and potentially affected their loneliness, we used the mean score of five items that refer to different facets of the change in respondent’s personal life (i.e., changes in daily routines, overall lifestyle, hobbies, traveling, financial prosperity; 7-point Likert scales, 1 = strongly disagree, 7 = strongly agree); we refer to this variable as “*personal COVID-19 restraints*”. In addition, we also measured our respondents’ “work-life COVID-19 restraints”; we did so with a single item that had three scale points (“no effect on job,” “some effects [e.g., home office],” and “can’t work at all”). We also included a number of consumer characteristics, namely a respondent’s self-reported demographics, in terms of age (1. “16–24 years old,” 2. “25–34 years old,” 3. “35–44 years old,” 4. “45–54 years old,” 5.”55 years or older”), gender (1. “male,” 2. “female”), education (1. “no education” or “lower secondary education,” 2. “middle school,” 3. “academic high school” or “academic degree”), during-lockdown employment status (1. “student,” 2. “employed,” 3. “unemployed,” 4. “retired”), and if the respondent was living alone during the surveyed lockdown.

We surveyed our respondents’ personality using single items for each of the Big Five personality traits based on the scale developed by Rammstedt et al. [48] (Extraversion: “I am outgoing and social”, Agreeableness: “I am entrusting and belief in the good in man,” Conscientiousness: “I handle all tasks thoroughly,” Neuroticism: “I easily get nervous and insecure,” Openness: “I am fanciful and have an active imagination”; all measured on a 7-point Likert scale, 1 = strongly disagree, 7 = strongly agree). We also measured respondents’ social media confidence based on Bright, Kleiser & Grau [49] (“I feel confident using digital social media.”; 7-point Likert scale, 1 = strongly disagree, 7 = strongly agree).

In Table 1, we list the items for all variables we included in our statistical analyses.

**Table 1:** Overview of used items.

Construct	Items	Scale
Media usage	*How many hours do you spend on an average weekday using the following media*:	In hours
	*Social media (e*.*g*., *Facebook*, *Twitter*, *Instagram etc*.*)*, *Online and offline Newspapers*, *Radio*, *Television*, *Blogs*, *Podcasts*	
Loneliness	*How often do you feel alone*?	7-point frequency (from 1 = never to 7 = very frequently)
	*How often do you feel lonely*?	
	*How often do you feel left out*?	
Face-to-face interaction	*How much time do you currently spend talking face-to-face in your private time*?	In hours
Video chat interaction	*How often do you currently use video telephony (e*.*g*., *Skype*, *WhatsApp calls*, *Zoom*, *etc*.*) for private communication*, *e*.*g*., *to talk to your friends*, *your acquaintances*, *or your family or to share private things*?	5-point intensity (from 1 = never to 5 = multiple times a day)
Personal COVID-19 restraints	*My daily routine has changed drastically*.	7-point agreement (from 1 = strongly disagree to 7 = strongly agree)
	*I had to majorly change my overall lifestyle*.	
	*I cannot pursue my hobbies anymore*.	
	*My travel habits have fundamentally changed*.	
	*My financial prosperity has deteriorated significantly*.	
Work-life COVID-19 restraints	*Has your daily work routine changed as a result of the coronavirus*?	Single choice between: No, it had no effect on my job., Yes, some effects [e.g., home office, social distance measures]., Yes, I cannot work at all.
Conscientiousness	*I handle all tasks thoroughly*.	7-point agreement (from 1 = strongly disagree to 7 = strongly agree)
Agreeableness	*I am entrusting and belief in the good in man*.	7-point agreement (from 1 = strongly disagree to 7 = strongly agree)
Extraversion	*I am outgoing and social*.	7-point agreement (from 1 = strongly disagree to 7 = strongly agree)
Neuroticism	*I easily get nervous and insecure*.	7-point agreement (from 1 = strongly disagree to 7 = strongly agree)
Openness	*I am fanciful and have an active imagination*.	7-point agreement (from 1 = strongly disagree to 7 = strongly agree)
Social media confidence	*I feel confident using digital social media*.	7-point agreement (from 1 = strongly disagree to 7 = strongly agree)
Age	*Please indicate your age*:	Single choice between: 16–24 years, 25–34 years, 35–44 years, 45–54 years, Older than 55 years
Female	*Please indicate your gender*:	Single choice between: Male, Female
Educational attainment	*Please select your highest degree of education*:	Single choice between: None, Lower secondary education, Middle school, Academic high school, Academic degree
Status of employment	*Please indicate your current status of employment*:	Single choice between: Student, Employed, Unemployed, Retired
Living alone	*Do you live in a household with other people*?	Single choice between: Yes, No

The empirical study was approved by the social science ethics commission of the School of Business and Economics at the University of Münster. Participants provided informed consent before being able to proceed to filling out the questionnaire.

### Sample

The total sample we analyze consists of two waves of responses from 825 people in Germany with a minimum age of 16 years. It is largely representative of the German population over the age of 16 in terms of age, gender, and education. Table 2 contrasts our sample composition with data from Germanys national census from 2019 [50].

**Table 2:** Demographic characteristics of respondents in comparison to German census.

	Sample	National census
Gender: Female (T0)	47.9%	48.8%
Gender: Male (T0)	52.1%	51.2%
Age: 16–24 (T0)	.4%	10.6%
Age: 25–34 (T0)	11.8%	15.3%
Age: 35–44 (T0)	14.9%	14.1%
Age: 45–54 (T0)	31.2%	16.5%
Age: >54 (T0)	41.7%	43.5%
Education: Low (T0)	13.0%	23.0%
Education: Medium (T0)	28.6%	36.0%
Education: High (T0)	58.4%	41.0%

By representing people older than 16 years we are able to provide insights for the age group constituting the vast majority on mainstream social media users (e.g., Facebook, Twitter, Instagram etc.); accounting for approximately 93% of their user base in 2021 [51–53].

In addition to the demographic characteristics of our final sample, Table 3 provides a descriptive overview of our main model variables, before and during the lockdown; namely loneliness, social media usage, other media usage, and face-to-face/video chat communication. The descriptive statistics indicate low overall levels of loneliness (mean_pre_: 2.69; mean_during_: 2.56) on an aggregate level, for both the pre-lockdown and lockdown survey. Notably, we even find *lower* average levels of loneliness during Germany’s initial lockdown. This rather surprising decrease in consumer loneliness during Germanys’ initial lockdown has also been noted by prior research [54–56] and is addressed further in the discussion section of this paper.

**Table 3:** Descriptive statistics of loneliness, media usage, and communication channels pre and during Germany’s initial lockdown.

	Minimum	Maximum	Mean	Standard deviation	N
Loneliness pre-lockdown (T1)	1	7	2.69	1.37	825
Loneliness during-lockdown (T2)	1	7	2.56	1.47	825
Social media usage pre-lockdown (T1)	0	16	.70	1.22	825
Social media usage during-lockdown (T2)	0	12	.73	1.21	825
Face-to-face communication pre-lockdown (T0)	0	20.3	2.49	2.60	825
Face-to-face communication during-lockdown (T2)	0	18	2.05	2.61	825
Video chat communication pre-lockdown (T0)	1	5	2.02	1.24	825
Video chat communication during-lockdown (T2)	1	5	2.07	1.34	825

With regards to social media usage, the self-reported average hours spent using social media marginally increased during the lockdown survey. However, a corresponding t-test indicated no significance of this change in the average usage of social media. With regards to people’s social interaction our descriptive statistics indicate that the level of face-to-face interaction declined on average during the lockdown (p < .01), as expected. However, the average amount of video chat communication did not significantly change.

### Empirical modeling

**Fig 3:**
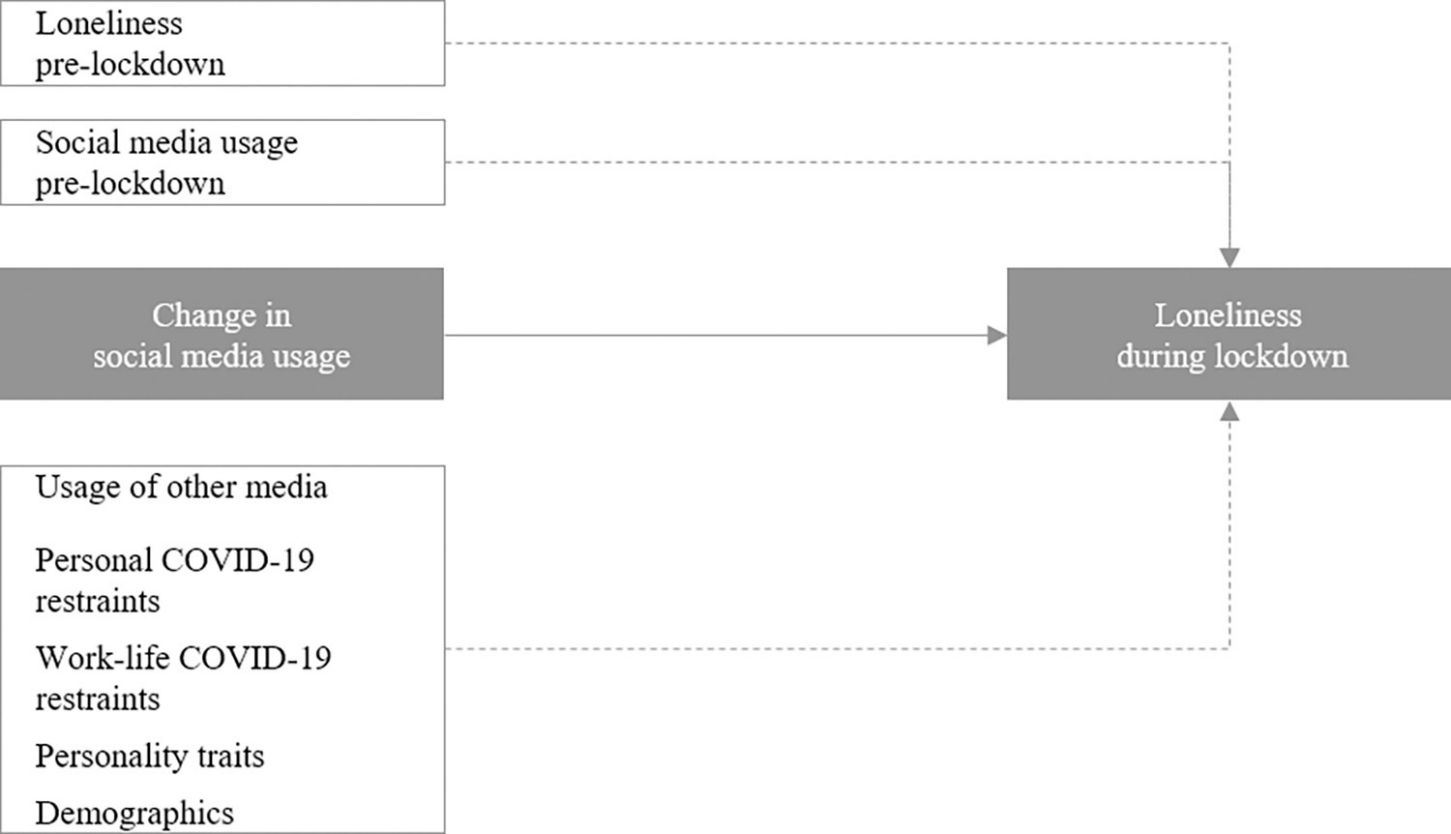
Empirical model of the main analysis.

To test our competing hypotheses, we use structural equation modeling (SEM) as implemented in AMOS 27. We prefer it over alternative econometric approaches such as multiple ordinary least square regressions because of its ability to include multi-item scales [57]. Fig 3 shows the empirical model employed within this research.

As we employ a longitudinal SEM that controls for pre-lockdown loneliness, we compared the corresponding configural, weak invariance and strong invariance model to determine whether our statistical model did indeed capture the argued change in people’s loneliness, our latent construct of interest. In doing so, the weak invariance model was constrained to equal factor loadings and the strong invariance model was constrained to equal factor loadings and equal intercepts. The comparison of these model specifications shows that the weak invariance model does not differ significantly from our configural model (*p =* .28), indicating that the construct of loneliness was captured analogously pre- and during the examined lockdown. Furthermore, all three specifications (configural, weak invariance, and strong invariance) indicate comparable effects in terms of size and significance, with the weak invariance model providing the strongest model fit in terms of root mean square error of approximation (RMSEA), comparative fit index (CFI) and the standardized root mean squared residual (SRMR). Hence, the invariance analyses indicate that the used statistical model indeed captures the change in our latent construct of interest, and we proceed to use the weak invariance model in our main analysis due to it providing the strongest model fit indices.

The supporting information contains further information on additional model specifications including the configural, weak invariance and strong invariance model as well as re-specifications that serve as robustness checks featuring different sets of covariates (see S1 File).

## Results

### Main analysis

The model fits the data well in terms of RMSEA (RMSEA = .037, RMSEA_Low_ = .030, RMSEA_high_ = .044), the CFI (.986) and SRMR (.017), with all indicators matching the respective thresholds for a good fit of the analyzed model [58]. In addition, fitting the model provides a chi-square of 198.13, with 93 degrees of freedom and a corresponding significance of less than 5%. The resulting significance of the chi-square test is not surprising as it is sensitive to larger sample sizes, leading to erroneous rejection of a good model fit in many such cases of larger sample sizes [59]. As all other model fit indices show a good or even very good fit, we conclude that our model fits the data adequately well.

Table 4 contains both the unstandardized (B) and standardized (β) parameter estimates for all variables as well as their levels of significance based on bootstrapped (n_bootstrap_ = 10.000) standard errors (SE), along with the squared multiple correlation (R^2^) of our outcome variable. The unstandardized coefficients B of all media usage variables can be compared intuitively as they rely on identical scales (i.e., usage hours), while the standardized coefficients ß allow a more general comparison of effect sizes across all incorporated variables.

**Table 4:** Structural equation model coefficients (weak invariance model).

		DV: Loneliness during lockdown				
Category	Variable	B		ß		SE
Social media	Change in use of SM (T2 -T1)	.131	*	.099	*	.040
usage	Baseline use of SM (T1)	.083	*	.067	*	.039
Other interaction	Change in F2F communication (T2 -T1)	-.057	**	-.113	**	.016
channels	Baseline F2F communication (T1)	-.044	*	-.074	*	.019
	Change in VC communication (T2 -T1)	-.012		-.011		.031
	Baseline VC communication (T1)	.009		.007		.037
Other media	Change in use of other media (T2 -T1)	.002		.008		.009
consumption	Baseline use of other media (T1)	.005		.017		.009
Controls	Personal COVID-19 restraints (T2)	.231	**	.217	**	.028
	Work-life COVID-19 restraints (T2)	-.026		-.010		.081
	Conscientiousness (T2)	-.037		-.029		.033
	Agreeableness (T2)	.065	**	.069	**	.024
	Extraversion (T2)	.025		.026		.027
	Neuroticism (T2)	.084	**	.092	**	.026
	Openness (T2)	-.055	*	-.058	*	.025
	Age (T0)	-.119	**	-.081	**	.044
	Female (T0)	.036		.012		.078
	Educational attainment (T0)	-.059		-.027		.058
	Unemployed (T2)	.122		.020		.167
	Retired (T2)	.261	*	.074	*	.120
	Living alone (T2)	.118		.035		.089
	Social media confidence (T2)	-.023		-.025		.026
	Loneliness (pre-lockdown) (T1)	.654	**	.614	**	.033

Note. R^2^ = .593. B = unstandardized coefficient; ß = standardized coefficient; SE = standard error; SM = social media; F2F = face-to-face; VC = video chat. Number of observations: 825

* p < .05

** p < .01 (two tailed)

The main result of our analysis is that during Germany’s initial COVID-19 lockdown, an increase in social media usage was associated with *higher*, not lower levels of loneliness (B: .131; p < .05). Thus, *H1A* is supported, hinting at a negative association between social media usage and people’s loneliness in times when face-to-face interaction is already restricted by external factors. Analogously, we reject *H1* as we find no negative association of social media and loneliness, which would support the compensation logic that social media usage may help people compensate for their situationally restricted face-to-face interactions underlying H1.

We additionally find that higher *baseline* social media usage is also associated with higher levels of loneliness (B: .083; p < .05). This association of our social media baseline is smaller in size and weaker in significance compared to our measure of change in social media usage. Nonetheless, both measures consistently hint at a negative relationship between social media usage and loneliness, but provide no support for potential social benefits offered by social media in times of social restrictions.

Regarding the additional model variables, we find that face-to-face communication is associated with lower levels of loneliness, but we do not find any significant direct association between video chat communication and loneliness. The consumption of media other than social media also is not significantly related to people’s loneliness. However, high levels of personal COVID-19 restraints are associated with higher loneliness, as could be expected, whereas COVID-19 restraints of work life show no statistical relation to loneliness.

Our results further suggest that demographics (e.g., age being a buffer against increased loneliness, whereas retirees are vulnerable to increased loneliness) and that some personality traits are related to people’s levels of loneliness during the lockdown. With respect to the latter, our findings add to the stream of literature linking personality traits to loneliness, both in “regular” (i.e., non-lockdown) settings [60] and in the extraordinary context of lockdowns [61–65]. Specifically, we find that neuroticism (B: .084; p < .01) and agreeableness (B: .065; p < .01) are associated with higher levels of loneliness, whereas openness (B: -.055; p < .05) is associated with lower levels of loneliness, and other traits (i.e., conscientiousness and extraversion) do not show significant associations in our analysis. While some of these results are in line with extant research (e.g., the association of neuroticism with higher levels of loneliness [61, 62]), some variations also exist, which we address in the subsequent discussion section.

Finally, robustness checks, including re-specifications of the SEM and separate ordinary least square estimations, show neither systematic nor substantial variations in the results with respect to the direction of the effects, the significance, or the approximated effect size (see S1 File). No re-specification or additional analyses indicate a positive association between social media usage and people’s loneliness during the considered lockdown, which would be supportive of the social value that gives the medium its name.

### Post-hoc analysis

In order to substantiate the insights of our main analysis and to test for additional indicators that support the crowding out effect that we argue underlies the uncovered positive association between social media usage and loneliness, we conducted a continuative post-hoc analysis with our panel dataset. Specifically, this post-hoc analysis focused on the interaction terms of the usage of “rich” interaction channels, namely face-to-face interactions as well as digital communication via video chats [33, 40, 66], along with social media during the lockdown period. Video chats via software such as Zoom have been found to enable higher levels of social presence, are considered richer media, and also tend to be more frequently used for social interaction with closer ties in non-occupational social interactions [37, 38]. Thus, using both social media and richer digital media such as video chats during a lockdown should indicate less substitution of valuable social interactions and consequently less loneliness. Consequently, significant interaction terms would be in line with the substitution mechanism that we argue could be the driver of the correlational effects our main analysis revealed and would be supportive of a directional effect of social media usage on loneliness.

**Fig 4:**
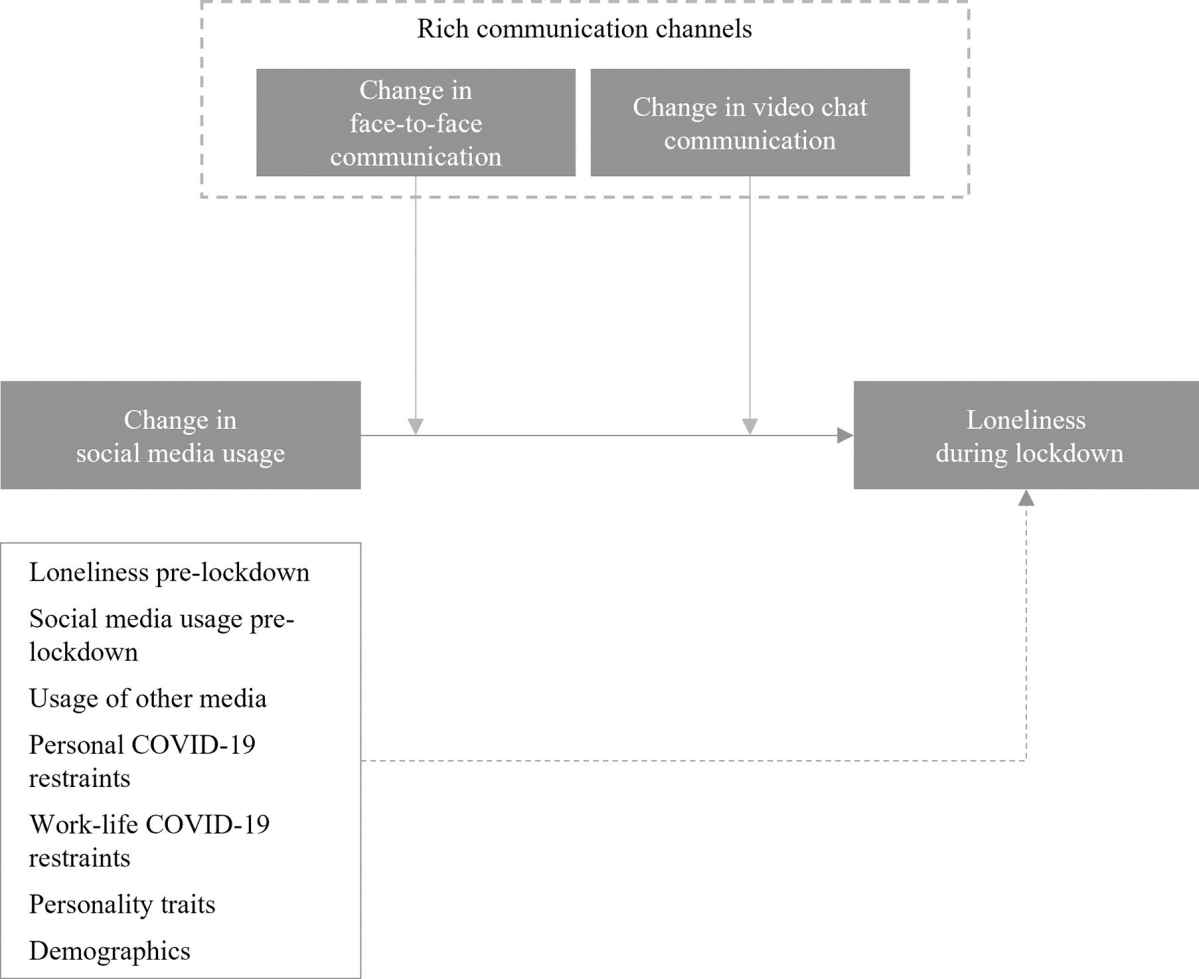
Post-hoc analysis.

To analyze whether richer media during a lockdown are indeed associated with a less pronounced link between social media usage and loneliness, we added two interaction terms to our model, both of which involve change in social media usage during the lockdown (i.e., the change in social media usage between lockdown and before). As shown in Fig 4, the first interaction term captures the interaction of change in social media usage and change in face-to-face communication during the lockdown (i.e., the change in face-to-face communication between lockdown and before). The second interaction term captures the interaction of change in social media usage and change in video chat communication during the lockdown (i.e., the change in video chat communication between lockdown and before). In addition to the interaction terms, our statistical approach also includes the direct effect of our postulated moderators, namely the change in face-to-face communication and the change in video chat communication.

Table 5 reports the corresponding standardized (β) parameter estimates for all variables as well as their levels of significance based on bootstrapped (n_bootstrap_ = 10.000) SE. In line with our reasoning, we find that an increase in video chat communication during the lockdown reduces the positive association of social media on loneliness (ß: -.088; p < .01), while an increase of face-to-face interaction during the lockdown does not significantly influence the link between social media and loneliness. We assume that video chats could be effectively used as an alternative to social media during the lockdown, while this was not the case with face-to-face communication which was extensively restricted by the decreed lockdown measures. Thus, these additional results suggest that “rich” communication can indeed dampen the positive association between social media and loneliness during a lockdown, but only if the compensatory communication channel is not facing restrictions.

**Table 5:** Post-hoc analysis: Structural equation model coefficients (weak invariance model).

		DV: Loneliness during lockdown		
Category	Variable	ß		SE
Social media	Change in use of SM (T2 –T1)	.078	*	.044
usage	Baseline use of SM (T1)	.072	*	.039
Other interaction	Change in F2F communication (T2 –T0)	-.112	**	.016
channels	Baseline F2F communication (T1)	-.077	*	.019
	Change VC communication (T2 –T0)	-.003		.031
	Baseline VC communication (T1)	.012		.037
Other media	Change in use of other media (T2 –T1)	.017		.009
consumption	Baseline use of other media (T1)	.024		.009
Controls	Personal COVID-19 restraints (T2)	.213	**	.028
	Work-life COVID-19 restraints (T2)	-.018		.081
	Conscientiousness (T2)	-.034		.033
	Agreeableness (T2)	.060	*	.024
	Extraversion (T2)	.031		.027
	Neuroticism (T2)	.093	**	.026
	Openness (T2)	-.054	*	.025
	Age (T0)	-.076	*	.044
	Female (T0)	.017		.077
	Educational attainment (T0)	-.022		.057
	Unemployed (T2)	.014		.167
	Retired (T2)	.071	*	.120
	Living alone (T2)	.035		.089
	Social media confidence (T2)	-.022		.025
	Loneliness (pre-lockdown) (T1)	.616	**	.033
Interactions	Change in use of SM x	.003		.010
	Change in F2F communication			
	Change in use of SM x	-.088	**	.018
	Change in VC communication			

Note. R^2^ = .600. ß = standardized coefficient; SE = standard error; SM = social media; F2F = face-to-face; VC = video chat. Number of observations: 825

*p < .05

** p < .01 (two tailed)

We interpret these additional results as further empirical support for our proposed displacement argument that we coined the “social media paradox”. Hence, we argue that our findings indicate that intense usage of video chats, as a media-rich digital alternative to restricted face-to-face communication, counter the undesirable substitution of rich physical communication by social media. Accordingly, we propose that the usage of social media is less harmful for people who increased their communication via richer digital channels during the lockdown. Thus, we argue that for people who use social media as a complementary communication channel rather than a substitute, intense social media usage should not be associated with increased levels of loneliness.

## Discussion

This study is the first to add longitudinal empirical data, including people’s baseline and changed behavior, to the ongoing debate about the relationship between social media usage and people’s loneliness in the specific context lockdowns, where face-to-face interactions were extensively restricted [30, 31, 39, 40]. We provide empirical evidence that in the six to eight-week window represented by our data collection, from before and during Germany’s nationwide lockdown in the spring of 2020, an increase in social media usage on average was associated with more, not less, loneliness. Additionally, our results indicate that people that generally use social media intensely and not only as a response to the lockdown measures, also experienced increased feelings of loneliness during the considered lockdown.

We attribute these correlational results to the mechanism explained by the “social media paradox” we propose in H1A. Following this line of argumentation, the presented results consistently hint at socially undesirable effects of social media usage in times of lockdown-induced social distancing. We argue that despite the reduction in face-to-face interactions, exchanges with social media “friends” not only did not compensate for the unavailability of social contacts in the physical realm, but even *deteriorated* the situation for the social media user in terms of experienced loneliness in our context.

Consistent with this line of arguments, our post-hoc analysis bolsters the notion of a “social media paradox” during the examined lockdown, as we find richer digital media to dampen social media’s association with higher levels of loneliness. These interaction effects further support the proposed directional relationship of the herein discussed effects, as they are in line with our conceptualized mechanism and antithetically would not be anticipated to show in the case where reverse causality would offer an alternative explanation.

Thus, we interpret the positive association between social media usage and loneliness, as well as the dampening effect of richer digital media on this relationship uncovered in our post-hoc analysis, as indicators that social media usage could crowd out more beneficial social interactions on other channels, hinting at a comparably limited social value for its users during Germany’s initial lockdown. This line of argument does not oppose a potential social value of social media, as indicated by previous research [23–26, 67], but rather suggests that the loss of more valuable social interactions, as postulated by our displacement logic, overcompensates for such value in the studied setting of Germany’s COVID-19 lockdown; reflected by a positive relationship between social media usage and loneliness.

Moreover, our study highlights that the relationship between social media usage and loneliness seems to be highly contextual, and the social value of social media seems to be highly contextual too. Relatedly, we attribute the existing conflicting findings on the relationship between social media usage and loneliness mainly to the differences in cultural (e.g., different countries) and situational (e.g., varying severity of lockdown measures) contexts across the study designs employed.

Overall, we interpret the negative association of social media usage and loneliness that we find for the initial COVID-19 lockdown in Germany, to be supportive of a “social media paradox” being at play during the studied lockdown that informs us about potential psychological downsides of social media usage. We thereby apply the logic of the “internet paradox” to social media usage displacing other richer digital communication channels and, most importantly, show its relevance in settings where face-to-face interaction is restricted. Notably, people’s baseline usage as well as their change in behavior are consistently associated with increased loneliness across all employed statistical analyses, giving no statistical evidence for a potential social value of social media usage during the considered lockdown. Thus, this research offers novel insights to the ongoing contradictory discussion of social media’s influence on people’s loneliness and the corresponding explanatory causal mechanism, by leveraging longitudinal data and employing additional analyses on the argued “social media paradox”. Specifically, our findings can be argued to be supportive of loneliness-enhancing effects of social media usage, as we attribute the negative relationship to an ineffectual displacement of more valuable social interaction via alternative digital and physical channels.

As we indicate in the results section, a related promising field for future research is the interplay between personality traits and people’s loneliness in different lockdown contexts. While our results are in line with extant research on neuroticism, they partially overlap with research on openness, and conscientiousness, but diverge from studies on extraversion. Specifically, while we and others [61] find that agreeableness is associated with higher loneliness, other studies find that agreeableness is negatively associated with loneliness [63, 65] or find no significant association between the two [62]. Similarly, current findings on the relationship between openness and loneliness also show heterogeneous effects, with some studies indicating a negative relationship [64, 65], in line with our observations, and other studies finding no significant link between the two [61–63]. For the relationship between conscientiousness and loneliness, the extant empirical insights also differ from each other, with some studies reporting positive [62], some negative [61] and some, including our study, nonsignificant effects [63]. Finally, our nonsignificant findings for the link between loneliness and extraversion diverge from the predominantly negative associations that other studies find [61, 62]. Interestingly, the more nuanced study by Landmann and Rohmann [63] indicates that the relationship of extraversion may differ across dimensions of loneliness. Specifically, they find a negative association of extraversion with the emotional and social dimensions of loneliness, but also a positive association with the physical loneliness dimension, indicating that studies using the aggregate loneliness measures may capture opposing effects that may cancel each other out in some lockdown settings.

Based on these very diverse findings across the extant research at hand, we argue that the varying contextual factors of these studies (e.g., phase of the pandemic or mandated lockdown restrictions) may impact the relationship of the considered personality traits and loneliness. First, the role of personality traits may change in different lockdown contexts as people’s perception of the social restrictions in place may be subject to change. Thus, the social benefits associated with extraversion, agreeableness, conscientiousness and openness in non-lockdown settings may be associated with increased perceptions of social loss in lockdown contexts [61, 62], where people have already internalized the social restrictions in place. Contrastingly, as our study is set in the very early phase of Germany’s initial COVID-19 lockdown, where the population did not report an overall increase in loneliness at the time, the relationship between people’s personality traits and loneliness may be different. Relatedly, Zacher and Rudolph [68] find a change in the direction of the relationship between some personality traits and people’s perceived stressfulness of the COVID-19 pandemic during different phases of Germany’s COVID-19 lockdowns. Hence, their findings support the notion that the relationship between personality traits and people’s well-being, including loneliness, may change in different lockdown contexts. However, as the current empirical insights on this matter are very limited, future research that sheds light on the differential role of personality traits for people’s loneliness during different lockdown contexts is needed.

Our findings should encourage individual users to reconsider their intense reliance on social media, both in general, but in particular during times of face-to-face restrictions. Notably, our correlational insights at hand do not support the notion that the extensive use of social media is per se an appropriate compensation strategy in times when physical social interaction is heavily restricted. Instead, based on our postulated “social media paradox,” we argue that people should use richer communication channels (e.g., video chats, but also social virtual reality alias the “metaverse” [69] and face-to-face conversations) if possible for social interaction to prevent higher levels of loneliness, and use social media rather as a supplement rather than a substitute for richer channels.

For policy makers, a directional interpretation of our correlational results cautions that the intense social media usage in the initial COVID-19 lockdowns could have had unwanted societal implications, fostering increasing loneliness among its users and in turn reducing the psychological well-being of social media users. Following this logic, future political mandates should account for the essential relevance of physical social interactions for people’s well-being and the differential effects of communication channels on loneliness. Besides adjusting future restrictions, policy makers may consider educating social media users (e.g., campaigns for diverse usage of social interaction channels) and providing accessible contact points for people experiencing loneliness, as well as safeguarding an adequate digital infrastructure allowing for richer digital channels to be properly used.

However, the limitations of this research need be considered, as is the case with most research. First, this paper addresses the initial consequences of social media usage in the case of a hitherto unprecedented event of a nationwide lockdown in Germany. During the considered initial lockdown, the practices of digital media were still evolving, celebrities were only starting lockdown support campaigns on Facebook and Instagram, and people who had never used video calls before were only adapting to this technology. Hence, our findings would need adjustment when translating them to subsequent lockdowns.

Second, generalizability may also be limited by the specific nature of the lockdown we study as well as the argued contextual nature of the examined relationship between social media usage and loneliness. Specifically, our data do not show negative *average* changes in people’s loneliness in our lockdown context. This result is consistent with the results from a representative German panel that studied people’s well-being during the COVID-19 pandemic [54]. Several reasons have been named for this, such as the relatively low mortality rate in Germany [55], stable medical supplies compared with other countries [24], the unusually good weather [56], and that the working population had more leisure and self-determination in working from home or in short-term jobs. Notably, our results show that social media usage was associated with increased levels of loneliness during a period when the general population was actually experiencing a reduction of loneliness. The generalizability of these findings to settings where loneliness on the societal level may have increased in response to an ongoing isolation due to long-term lockdown measures is uncertain.

Third, our empirical study relies on self-reported measures that, although commonly used within research related to social media [70–72], may be subject to individual biases and hence produce measurement errors [73]. Interestingly, current literature suggests that the predictive validity of self-reported measures on social media usage have a comparative predictive validity to digital trace measures nonetheless [73], which hints at the meaningfulness of the herein presented results. However, future research could address social media effects using digital trace measures to avoid measurement error in the first place and contribute even more robust insights into this matter.

Fourth, we have only surveyed our personality controls during the considered lockdown period, but not before. We are hence unable to rule out biased self-reports due to the experienced lockdown situation. However, prior research indicates that self-reported Big Five measures are rather stable and consistent over time [74]. As the time span between pre- and during lockdown data is less than two months, we assume possible changes in self-reports of such stable personality traits to be minor. Relatedly, multiple scholars have also used comparable measures that were surveyed during a lockdown situation as predictors of psychological responses to the respective COVID-19 lockdown [62, 75, 76].

Fifth, as we use survey data, we cannot establish causality through our empirical insights but rather interpret the reported correlational association between social media usage and loneliness as a result of our proposed causal mechanism between the two.

Finally, the sample is largely representative of Germany’s population older than 16 years of age, but it does not include people younger than that. It is unclear whether our results hold for this younger age group, and we refrain from speculation. Still, the share of the population represented by our sample constitutes more than 84% of the German population [50] as well as more than 93% of the user base of mainstream social media sites (e.g., Facebook, Twitter, Instagram etc.). Related, we only analyze data from people in Germany, which carries limitations for the broader generalizability of our findings to other countries.

## Supporting information

S1 File Additional statistical analyses. Describes all additional statistical analyses referred to in the manuscript.
(PDF)

S2 File Code used for statistical analyses. Provides the used code (Amos 27 and R) for the main analyses referred to in the manuscript.
(PDF)

S1 Data (SAV)
