# Supplementary material for: When the antidote is the poison: Investigating the relationship between people’s social media usage and loneliness when face-to-face communication is restricted
Source: PLoS One. 2024 Feb 9;19(2):e0296423. doi: 10.1371/journal.pone.0296423 (PMC10857570; doi:10.1371/journal.pone.0296423)
Supplement: S1 File — Describes all additional statistical analyses referred to in the manuscript. (PDF) [file pone.0296423.s001.pdf]

S1 Additional statistical analyses

S 1.1 Invariance analysis

Since we employ a longitudinal SEM that controls for pre-lockdown loneliness, we compared the corresponding configural, weak invariance and strong invariance model to determine whether our statistical approach did indeed capture the argued change in our latent construct of interest; consumers' loneliness. Tables S1 – S3 grant an overview for both the unstandardized (B) and standardized (β) parameter estimates for all variables as well as their levels of significance based on bootstrapped ($n_{bootstrap}$=10.000) standard errors (SE), for the configural, weak invariance and strong invariance model.

Table S1. Structural equation model coefficients (configural model).

		DV: Loneliness during lockdown		
**Category**	**Variable**	**B**	**ß**	**SE**
Social media usage	Change in use of SM (T2 -T1)	.132 *	.100 *	.040
	Normal use of SM (T1)	.084 *	.067 *	.039
Other interaction channels	Change in F2F communication (T2 -T1)	-.057 **	-.114 **	.016
	Normal F2F communication (T1)	-.044 *	-.075 *	.019
	Change in VC communication (T2 -T1)	-.011	-.010	.031
	Normal VC communication (T1)	.009	.007	.037
Other media consumption	Change in use of other media (T2 -T1)	.002	.008	.009
	Normal use of other media (T1)	.005	.017	.009
Controls	Personal COVID-19 restraints (T2)	.232 **	.218 **	.029
	Work-life COVID-19 restraints (T2)	-.026	-.010	.082
	Conscientiousness (T2)	-.038	-.030	.034
	Agreeableness (T2)	.065 **	.069 **	.024
	Extraversion (T2)	.024	.025	.027
	Neuroticism (T2)	.085 **	.092 **	.026
	Openness (T2)	-.055	-.057	.025
	Age (T0)	-.119 **	-.081 **	.044
	Female (T0)	.037	.012	.078
	Educational attainment (T0)	-.057	-.027	.058
	Unemployed (T2)	.122	.020	.168
	Retired (T2)	.262 *	.074 *	.121
	Living alone (T2)	.120	.035	.090
	Social media confidence (T2)	-.023	-.026	.026
	Loneliness (pre-lockdown) (T1)	.657 **	.614 **	.035

Note. $R^2 = .593$. *B= unstandardized coefficient; ß= standardized coefficient; SE = standard error; SM = social media; F2F = face-to-face; VC = video chat. Number of observations: 825.* * $p < .05$; ** $p < .01$ *(two tailed)*

Table S2. Structural equation model coefficients (weak invariance model).

		DV: Loneliness during lockdown		
**Category**	**Variable**	B	ß	SE
Social media usage	Change in use of SM (T2 -T1)	.131 *	.099 *	.040
	Baseline use of SM (T1)	.083 *	.067 *	.039
Other interaction channels	Change in F2F communication (T2 -T1)	-.057 **	-.113 **	.016
	Baseline F2F communication (T1)	-.044 *	-.074 *	.019
	Change in VC communication (T2 -T1)	-.012	-.011	.031
	Baseline VC communication (T1)	.009	.007	.037
Other media consumption	Change in use of other media (T2 -T1)	.002	.008	.009
	Baseline use of other media (T1)	.005	.017	.009
Controls	Personal COVID-19 restraints (T2)	.231 **	.217 **	.028
	Work-life COVID-19 restraints (T2)	-.026	-.010	.081
	Conscientiousness (T2)	-.037	-.029	.033
	Agreeableness (T2)	.065 **	.069 **	.024
	Extraversion (T2)	.025	.026	.027
	Neuroticism (T2)	.084 **	.092 **	.026
	Openness (T2)	-.055 *	-.058 *	.025
	Age (T0)	-.119 **	-.081 **	.044
	Female (T0)	.036	.012	.078
	Educational attainment (T0)	-.059	-.027	.058
	Unemployed (T2)	.122	.020	.167
	Retired (T2)	.261 *	.074 *	.120
	Living alone (T2)	.118	.035	.089
	Social media confidence (T2)	-.023	-.025	.026
	Loneliness (pre-lockdown) (T1)	.654 **	.614 **	.033

Note. $R^2 = .593$. *B= unstandardized coefficient; ß= standardized coefficient; SE = standard error; SM = social media; F2F = face-to-face; VC = video chat. Number of observations: 825. * $p < .05$; ** $p < .01$ (two tailed)*

Table S3. Structural equation model coefficients (strong invariance model).

Category	Variable	DV: Loneliness during lockdown B	ß	SE
Social media usage	Change in use of SM (T2 -T1)	.129 *	.098 *	.040
	Normal use of SM (T1)	.077 *	.062 *	.039
Other interaction channels	Change in F2F communication (T2 -T1)	-.057 **	-.113 **	.016
	Normal F2F communication (T1)	-.043 *	-.074 *	.019
	Change in VC communication (T2 -T1)	-.009	-.009	.031
	Normal VC communication (T1)	.015	.012	.037
Other media consumption	Change in use of other media (T2 -T1)	.001	.005	.009
	Normal use of other media (T1)	.003	.013	.009
Controls	Personal COVID-19 restraints (T2)	.224 **	.211 **	.028
	Work-life COVID-19 restraints (T2)	-.043	-.017	.080
	Conscientiousness (T2)	-.054	-.043	.031
	Agreeableness (T2)	.062 **	.066 **	.024
	Extraversion (T2)	.021	.022	.027
	Neuroticism (T2)	.074 **	.080 **	.025
	Openness (T2)	-.059 *	-.062 *	.025
	Age (T0)	-.149 **	-.102 **	.037
	Female (T0)	.035	.012	.078
	Educational attainment (T0)	-.089	-.042	.053
	Unemployed (T2)	.087	.015	.165
	Retired (T2)	.254 *	.072 *	.120
	Living alone (T2)	.110	.032	.089
	Social media confidence (T2)	-.026	-.029	.025
	Loneliness (pre-lockdown) (T1)	.651 **	.612 **	.033

*Note. R^2 = .592. B= unstandardized coefficient; ß= standardized coefficient; SE = standard error; SM = social media; F2F = face-to-face; VC = video chat. Number of observations: 825. * p < .05; ** p < .01 (two tailed)*

All specifications provide a sufficient fit in terms of RMSEA ($\text{RMSEA}^{\text{configural}}$ = .037, $\text{RMSEA}^{\text{configural}}_{\text{Low}}$= .030, $\text{RMSEA}^{\text{configural}}_{\text{high}}$= .044; $\text{RMSEA}^{\text{weak invariance}}$ = .037, $\text{RMSEA}^{\text{weak invariance}}_{\text{Low}}$= .030, $\text{RMSEA}^{\text{weak invariance}}_{\text{high}}$= .044; $\text{RMSEA}^{\text{strong invariance}}$ = .038, $\text{RMSEA}^{\text{strong invariance}}_{\text{Low}}$= .031, $\text{RMSEA}^{\text{strong invariance}}_{\text{high}}$= .045), the CFI ($\text{CFI}^{\text{congifural}}$ = .986; $\text{CFI}^{\text{weak invariance}}$ = .986; $\text{CFI}^{\text{strong invariance}}$ = .984) and SRMR ($\text{SRMR}^{\text{configural}}$ = .0169; $\text{SRMR}^{\text{weak invariance}}$ = .0169; $\text{SRMR}^{\text{strong invariance}}$ = .0170), with all indicators matching the respective thresholds for a good fit [1].

Notably, the coefficients and corresponding standard errors only differ marginally across the different specifications of our model, which indicates the robustness of our reported statistical associations. Relatedly, the model comparison between the configural and

weak invariance model do not indicate a significant differentiation between the two models (*p*=.28). Therefore, we are confident that that the construct of loneliness was captured analogously pre- and during the examined lockdown.

However, as the model fit for the weak invariance model is slightly higher, than for the strong invariance model we used it to report our analyses in the manuscript (both the main analysis as well as the post-hoc analysis).

S 1.2 Confirmatory factor analysis

To ensure an adequate item fit of our multi-item measures, we conducted a confirmatory factor analysis with AMOS 27; Fig S1 shows the standardized path coefficients. The results indicate sufficient factor loadings for all used items being > .075 [2]. Thus, the reflective scales did not have to be refined for further analysis.

Fig S1. Standardized estimates of CFA using Amos 27.

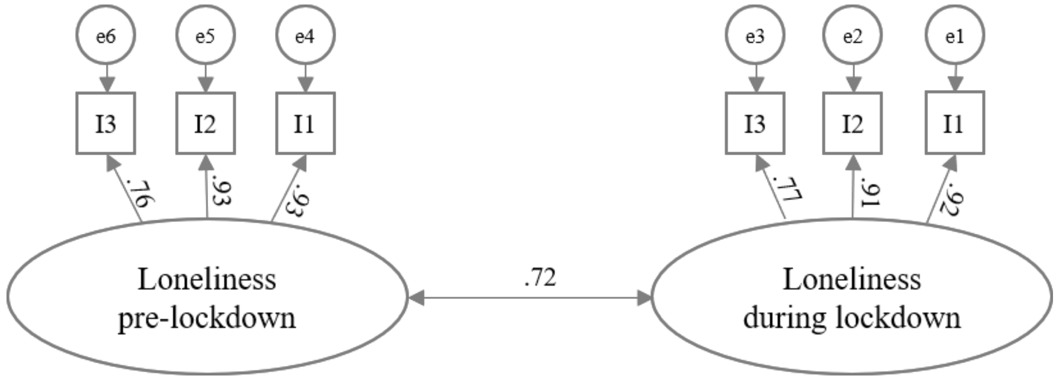

S 1.3 OLS regression as robustness check

In addition to our structural equation modeling (SEM) reported in the manuscript, we conducted ordinary least square (OLS) regression, using R, mirroring the SEM we conducted. The OLS regression was constructed analogously to the main SEM model and yields results in terms of significance levels and coefficient size; not indicating any systematic deviation to our main analysis reported in the manuscript. The unstandardized coefficients (B) and corresponding p-values can be seen in Table S4. Furthermore, we calculated variance

inflation factors (VIF) for the regression to assess possible multicollinearity problems; all resulting VIFs were below 2 (see Table S4), indicating that multicollinearity does not bias the results in any concerning way.

Table S4. Coefficients for main model estimated with OLS.

		**DV: Loneliness during lockdown**		
**Category**	**Variable**	B	SE	VIF
Social media usage	Change in use of SM (T2 -T1)	.114 **	.037	1.499
	Normal use of SM (T1)	.089 *	.037	1.646
Other interaction channels	Change in F2F communication (T2 -T1)	-.053 **	.015	1.642
	Normal F2F communication (T1)	-.043 *	.017	1.716
	Change in VC communication (T2 -T1)	-.005	.029	1.452
	Normal VC communication (T1)	.014	.035	1.550
Other media consumption	Change in use of other media (T2 -T1)	.003	.009	1.686
	Normal use of other media (T1)	.005	.008	1.792
Controls	Personal COVID-19 restraints (T2)	.224 **	.026	1.193
	Work-life COVID-19 restraints (T2)	-.008	.076	1.753
	Conscientiousness (T2)	-.033	.031	1.161
	Agreeableness (T2)	.058 **	.022	1.087
	Extraversion (T2)	.016	.025	1.376
	Neuroticism (T2)	.087 **	.024	1.313
	Openness (T2)	-.046 *	.023	1.174
	Age (T0)	-.111 **	.041	1.506
	Female (T0)	.033	.072	1.083
	Educational attainment (T0)	-.065	.053	1.208
	Unemployed (T2)	.132	.155	1.318
	Retired (T2)	.260 *	.112	1.940
	Living alone (T2)	.103	.082	1.132
	Social media confidence (T2)	-.029	.024	1.339
	Loneliness (pre-lockdown) (T1)	.624 **	.030	1.421

Note. $R^2 = .553$. *B= unstandardized coefficient; SE = standard error, VIF = variance inflation factor; SM = social media; F2F = face-to-face; VC = video chat. Number of observations: 825. * p < .05; ** p < .01 (two tailed).*

S 1.4 Alternative SEM specifications as robustness checks

To test the robustness of our effects we carried out additional analysis using a different set of covariates (also reported in Table S5). Instead of using time-invariant personality traits and demographics as controls, we used only time-varying controls for other media usage. This alternative specification yields similar results to those from the main analysis, as reported in the manuscript (same direction of the effects, similar p-values); the corresponding standardized coefficients are in Table S5. The coefficients of this alternative specification even indicate a slightly stronger undesirable association of social media usage

and loneliness during Germany's initial lockdown. Furthermore, the model fit is still adequate ($RMSEA^{Alternative}$: .022; $CFI^{Alternative}$: .997).

Table S5. SEM coefficients for main and alternative specification (weak invariance model).

		DV: Loneliness during lockdown			
		Main model		Alternative	
**Category**	**Variable**	ß	SE	ß	SE
Social media usage	Change in use of SM (T2 -T1)	.099 *	.040	.127 *	.041
	Normal use of SM (T1)	.067 *	.039	.090 **	.039
Other interaction channels	Change in F2F communication (T2 -T1)	-.113 **	.016	-.133 **	.016
	Normal F2F communication (T1)	-.074 *	.019	-.078 *	.019
	Change in VC communication (T2 -T1)	-.011	.031	.030	.033
	Normal VC communication (T1)	.007	.037	.061 *	.038
Other media consumption	Change in use of other media (T2 -T1)	.008	.009	.000	.010
	Normal use of other media (T1)	.017	.009	.030	.009
Controls	Personal COVID-19 restraints (T2)	.217 **	.028		
	Work-life COVID-19 restraints (T2)	-.010	.081		
	Conscientiousness (T2)	-.029	.033		
	Agreeableness (T2)	.069 **	.024		
	Extraversion (T2)	.026	.027		
	Neuroticism (T2)	.092 **	.026		
	Openness (T2)	-.058 *	.025		
	Age (T0)	-.081 **	.044		
	Female (T0)	.012	.078		
	Educational attainment (T0)	-.027	.058		
	Unemployed (T2)	.020	.167		
	Retired (T2)	.074 *	.120		
	Living alone (T2)	.035	.089		
	Social media confidence (T2)	-.025	.026		
	Loneliness (pre-lockdown) (T1)	.614 **	.033	.692 **	.030

Note. $R^2_{Main\ model} = .593$; $R^2_{Alternative} = .520$. *ß= standardized coefficient; SE = standard error; SM = social media; F2F = face-to-face; VC = video chat. Number of observations: 825. *p < .05. ** p < .01 (two tailed).*

S 1.5 Post-hoc analyses

As our main analysis as well as our robustness checks indicate a socially undesirable effect of social media usage in times of pandemic-induced social distancing, we carried out additional post-hoc analyses with our panel dataset to better the understanding of this effect. Following the reasoning of H1b, the undesirable effect of social media usage should be contingent on the magnitude of displaced "richer" interaction. Hence, high usage levels of

"rich" interaction channels should dampen the negative effect of social media on loneliness during Germany's initial lockdown.

To assess this, we ran additional analyses focusing on the interaction terms of consumers' usage of "rich" interaction channels and their respective social media usage. Specifically, we added two interaction terms both involving abnormal social media usage during the lockdown (i.e., the change in social media usage between lockdown and before): one with abnormal face-to-face communication during the lockdown (i.e., the change in face-to-face communication between lockdown and before) and the other with abnormal video chat communication during the lockdown (i.e., the change in video chat communication between lockdown and before).

Table S6 reports the standardized (β) parameter estimates for all variables as well as their levels of significance based on bootstrapped ($n_{bootstrap}$=10.000) standard errors (SE) for this specification.

Table S6. SEM coefficients for main and alternative post-hoc specifications (weak invariance model).

		DV: Loneliness during lockdown			
		Main model		Alternative	
**Category**	**Variable**	ß	SE	ß	SE
Social media usage	Change in use of SM (T2 -T1)	.078 *	.044	.098 **	.046
	Normal use of SM (T1)	.072 *	.039	.097 **	.039
Other interaction channels	Change in F2F communication (T2 -T1)	-.112 **	.016	-.130 **	.016
	Normal F2F communication (T1)	-.077 *	.019	-.080 *	.019
	Change in VC communication (T2 -T1)	-.003	.031	.039	.032
	Normal VC communication (T1)	.012	.037	.066 *	.038
Other media consumption	Change in use of other media (T2 -T1)	.017	.009	.011	.010
	Normal use of other media (T1)	.024	.009	.039	.003
Controls	Personal COVID-19 restraints (T2)	.213 **	.028		
	Work-life COVID-19 restraints (T2)	-.018	.081		
	Conscientiousness (T2)	-.034	.033		
	Agreeableness (T2)	.060 *	.024		
	Extraversion (T2)	.031	.027		
	Neuroticism (T2)	.093 **	.026		
	Openness (T2)	-.054 *	.025		
	Age (T0)	-.076	.044		
	Female (T0)	.017	.077		
	Educational attainment (T0)	-.022	.057		
	Unemployed (T2)	.014	.167		
	Retired (T2)	.071 *	.120		
	Living alone (T2)	.035	.089		
	Social media confidence (T2)	-.022	.025		
	Loneliness (pre-lockdown) (T1)	.616 **	.033	.691 **	.030
Interactions	Change in use of SM x Change in F2F communication	.003	.010	.011	.011
	Change in use of SM x Change in VC communication	-.088 **	.018	-.103 **	.020

Note. $R^2_{Main\ model} = .600$ $R^2_{Alternative} = .530$. *ß= standardized coefficient; SE = standard error; SM = social media; F2F = face-to-face; VC = video chat. Number of observations: 825.* $*p < .05$. $**p < .01$ *(two tailed)*

The model fit indices for the post-hoc analyses indicate overall good model fits in terms of root mean square error ($RMSEA_{MainModel}$:.035, $RMSEA_{Alternative}$: .019) and comparative fit index ($CFI_{MainModel}$: .987, $CFI_{Alternative:}$ .998). Analogue to our main analysis reported in the manuscript the estimates for all variables as well as their levels of significance are based on bootstrapped standard errors ($n_{bootstrap}$= 10.000) and the included interactions are mean-centered and thus allow for an intuitive interpretation of the conditional main effects of abnormal social media usage.

The main result of this post-hoc analysis is the significant dampening effect of increased abnormal communication via video chats during the lockdown (ß: -.088**) on the undesirable effect of social media usage on loneliness. Notably, the increase of face-to-face interaction during the lockdown is not associated with dampening effects on the undesirable main effect of social media on loneliness. We speculate that video chats could be effectively used as an alternative to social media during the lockdown, while this was not the case with face-to-face communication which was restricted by the decreed lockdown measures. Thus, "rich" communication can indeed dampen the loneliness-enhancing impact of social media usage during a lockdown, but only if the compensatory communication channel is not facing restrictions.

To test the robustness of these interaction effects we again carried out an additional analysis using a different set of covariates (also reported in Table S6), again including only time-varying controls for other media usage instead of the time-invariant consumer traits and demographics we use as controls in the main model. Our alternative SEM analysis provides similar results with regards to coefficients and standard errors and does not hint at any systematic deviations.

S 1.6 References – SI 1

1. Hu L-t, Bentler PM. Cutoff criteria for fit indexes in covariance structure analysis: Conventional criteria versus new alternatives. Struct Equ Modeling. 1999; 6:1–55.

2. Hair J, Black W, Babin B, Anderson R. Multivariate data analysis. 8th ed. Boston: Cengage; 2018.
