# Supplementary material for: When the antidote is the poison: Investigating the relationship between people’s social media usage and loneliness when face-to-face communication is restricted
Source: PLoS One. 2024 Feb 9;19(2):e0296423. doi: 10.1371/journal.pone.0296423 (PMC10857570; doi:10.1371/journal.pone.0296423)
Supplement: S2 File — Provides the used code (Amos 27 and R) for the main analyses referred to in the manuscript. (PDF) [file pone.0296423.s002.pdf]

S2 Code used for statistical analyses

S 2.1 R-code used for OLS regression

```
####################################################################
############## Load and install used packages ############
#install.packages("readr")
library(readr)
#install.packages("haven")
library(haven)
#install.packages("jtools")
library(jtools)
#install.packages("interactions")
library(interactions)
#install.packages("sandwich")
library(sandwich)
#install.packages("car")
library(car)
####################################################################
############## Load local data (has to be adjusted to where data is located) ############
############################################################
setwd("XXXXX") # has to be adjusted to current data repository
Data_OLS <- read_sav(file = Minimal dataset.sav")
####################################################################
################ Building Containers for relevant variables  #############
############################################################
dv     <- c("Loneliness_mean_w2")
dv_lag <- c("Loneliness_mean_w1")

iv <- c("SM_Total_change_c + SM_Total_w1_c")

controls <- c("commun_FacetoFace_change_c","commun_FacetoFace_w0_c",
"commun_VideoChats_change_c", "commun_VideoChats_w0_c",
"OtherMedia_Total_change", "OtherMedia_Total_w1", "CorRestrLeis_w2",
"CorRestrOcc_w2", "Conscientiousness_w2", "Agreeableness_w2","Extraversion_w2",
"Neuroticism_w2", "Openness_w2", "Age_w0", "Female_w0", "Education_w0",
"Unemployed_dummy_w2",Retired_dummy_w2","NoFlatmates_w2","Social_Media_
Confidence_w2")
####################################################################
################ Estimate OLS and print output as well as VIF ################
############################################################
regression_formula <- paste(dv,
            paste(c(iv,controls,dv_lag),collapse=" + "),  sep=" ~ ")

#estimate the ols
estimation <- lm(as.formula(regression_formula),data = Data_OLS)
#print output
```

```
summary(estimation)
#print corresponding vifs
vif(estimation)
```

S 2.2 Amos syntax main model

```
Loneliness_I1_w1 = (int_1) + (1) Loneliness_pl + (1) e2
Loneliness_I1_w2 = (int_2) + (1) e5 + (1) Loneliness_w2
Loneliness_I2_w1 = (int_3) + (w_2) Loneliness_pl + (1) e3
Loneliness_I2_w2 = (int_4) + (w_4) Loneliness_w2 + (1) e6
Loneliness_I3_w1 = (int_5) + (1) e4 + (w_3) Loneliness_pl
Loneliness_I3_w2 = (int_6) + (1) e7 + (w_6) Loneliness_w2

Loneliness_w2 = (0) +  (b1_2) SM_Total_change + (b2_2) SM_Total_w1 + (b7_2)
OtherMedia_Total_change + (b8_2) OtherMedia_Total_w1  + (b3_2)
Commun_FacetoFace_change_c + (b4_2) Commun_FacetoFace_w0_c + (b5_2)
Commun_VideoChats_change_c + (b6_2) Commun_VideoChats_w0_c + (b2214_2)
CorRestrLeis_w2 + (b15_2) CorRestrOcc_w2 +(b11_2) Conscientiousness_w2 + (b12_2)
Agreeableness_w2 + (b13_2) Extraversion_w2 + (b14_2) Neuroticism_w2 +(b1423_2)
Openness_w2 + (b16_2) Age +   (b17_2) Female + (b18_2) Education + (b19_2)
Unemployed_dummy(b20_2) Retired_dummy + (b21_2) NoFlatmates +(b22_2)
Social_Media_ Confidence _w2 + (b23_2) Loneliness_pl + (1) e1

CorRestrLeis_w2 <> CorRestrOcc_w2 (ccc1_2)
CorRestrOcc_w2 <> SM_Total_w1 (ccc2_2)
CorRestrOcc_w2 <> SM_Total_change (ccc3_2)
OtherMedia_Total_w1 <> CorRestrOcc_w2 (ccc4_2)
CorRestrOcc_w2 <> OtherMedia_Total_change (ccc5_2)
Education <> CorRestrOcc_w2 (ccc6_2)
Extraversion_w2 <> CorRestrOcc_w2 (ccc7_2)
Agreeableness_w2 <> CorRestrOcc_w2 (ccc8_2)
Conscientiousness_w2 <> CorRestrOcc_w2 (ccc9_2)
Neuroticism_w2 <> CorRestrOcc_w2 (ccc10_2)
Openness_w2 <> CorRestrOcc_w2 (ccc11_2)
Age <> CorRestrOcc_w2 (ccc12_2)
Unemployed_dummy <> CorRestrOcc_w2 (ccc13_2)
Retired_dummy <> CorRestrOcc_w2 (ccc14_2)
Female <> CorRestrOcc_w2 (ccc15_2)
CorRestrLeis_w2 <> SM_Total_w1 (ccc16_2)
CorRestrLeis_w2 <> SM_Total_change (ccc17_2)
OtherMedia_Total_w1 <> CorRestrLeis_w2 (ccc18_2)
OtherMedia_Total_change <> CorRestrLeis_w2 (ccc19_2)
Education <> CorRestrLeis_w2 (ccc20_2)
CorRestrLeis_w2 <> Extraversion_w2 (ccc21_2)
CorRestrLeis_w2 <> Agreeableness_w2 (ccc22_2)
Conscientiousness_w2 <> CorRestrLeis_w2 (ccc23_2)
Neuroticism_w2 <> CorRestrLeis_w2 (ccc24_2)
Openness_w2 <> CorRestrLeis_w2 (ccc25_2)
CorRestrLeis_w2 <> Age (ccc26_2)
CorRestrLeis_w2 <> Unemployed_dummy (ccc27_2)
CorRestrLeis_w2 <> Retired_dummy (ccc28_2)
CorRestrLeis_w2 <> Female (ccc29_2)
SM_Total_w1 <> SM_Total_change (ccc30_2)
```

```
SM_Total_w1 <> OtherMedia_Total_w1 (ccc31_2)
SM_Total_w1 <> OtherMedia_Total_change (ccc32_2)
Education <> SM_Total_w1 (ccc33_2)
Extraversion_w2 <> SM_Total_w1 (ccc34_2)
Agreeableness_w2 <> SM_Total_w1 (ccc35_2)
Conscientiousness_w2 <> SM_Total_w1 (ccc36_2)
Neuroticism_w2 <> SM_Total_w1 (ccc37_2)
Openness_w2 <> SM_Total_w1 (ccc38_2)
Age <> SM_Total_w1 (ccc39_2)
Unemployed_dummy <> SM_Total_w1 (ccc40_2)
Retired_dummy <> SM_Total_w1 (ccc41_2)
Female <> SM_Total_w1 (ccc42_2)
SM_Total_change <> OtherMedia_Total_w1 (ccc43_2)
SM_Total_change <> OtherMedia_Total_change (ccc44_2)
Education <> SM_Total_change (ccc45_2)
Extraversion_w2 <> SM_Total_change (ccc46_2)
Agreeableness_w2 <> SM_Total_change (ccc47_2)
Conscientiousness_w2 <> SM_Total_change (ccc48_2)
Neuroticism_w2 <> SM_Total_change (ccc49_2)
Openness_w2 <> SM_Total_change (ccc50_2)
Age <> SM_Total_change (ccc51_2)
Unemployed_dummy <> SM_Total_change (ccc52_2)
Retired_dummy <> SM_Total_change (ccc53_2)
Female <> SM_Total_change (ccc54_2)
OtherMedia_Total_change <> OtherMedia_Total_w1 (ccc55_2)
OtherMedia_Total_w1 <> Education (ccc56_2)
OtherMedia_Total_w1 <> Extraversion_w2 (ccc57_2)
OtherMedia_Total_w1 <> Agreeableness_w2 (ccc58_2)
OtherMedia_Total_w1 <> Conscientiousness_w2 (ccc59_2)
OtherMedia_Total_w1 <> Neuroticism_w2 (ccc60_2)
OtherMedia_Total_w1 <> Openness_w2 (ccc61_2)
OtherMedia_Total_w1 <> Age (ccc62_2)
OtherMedia_Total_w1 <> Unemployed_dummy (ccc63_2)
OtherMedia_Total_w1 <> Retired_dummy (ccc64_2)
OtherMedia_Total_w1 <> Female (ccc65_2)
OtherMedia_Total_change <> Education (ccc66_2)
OtherMedia_Total_change <> Extraversion_w2 (ccc67_2)
OtherMedia_Total_change <> Agreeableness_w2 (ccc68_2)
OtherMedia_Total_change <> Conscientiousness_w2 (ccc69_2)
OtherMedia_Total_change <> Neuroticism_w2 (ccc70_2)
OtherMedia_Total_change <> Openness_w2 (ccc71_2)
OtherMedia_Total_change <> Age (ccc72_2)
OtherMedia_Total_change <> Unemployed_dummy (ccc73_2)
OtherMedia_Total_change <> Retired_dummy (ccc74_2)
OtherMedia_Total_change <> Female (ccc75_2)
Education <> Extraversion_w2 (ccc76_2)
Education <> Agreeableness_w2 (ccc77_2)
Education <> Conscientiousness_w2 (ccc78_2)
Education <> Neuroticism_w2 (ccc79_2)
Education <> Openness_w2 (ccc80_2)
```

147 Education <> Age (ccc81_2)
148 Education <> Unemployed_dummy (ccc82_2)
149 Education <> Retired_dummy (ccc83_2)
150 Education <> Female (ccc84_2)
151 Agreeableness_w2 <> Extraversion_w2 (ccc85_2)
152 Conscientiousness_w2 <> Extraversion_w2 (ccc86_2)
153 Neuroticism_w2 <> Extraversion_w2 (ccc87_2)
154 Openness_w2 <> Extraversion_w2 (ccc88_2)
155 Age <> Extraversion_w2 (ccc89_2)
156 Unemployed_dummy <> Extraversion_w2 (ccc90_2)
157 Retired_dummy <> Extraversion_w2 (ccc91_2)
158 Female <> Extraversion_w2 (ccc92_2)
159 Conscientiousness_w2 <> Agreeableness_w2 (ccc93_2)
160 Neuroticism_w2 <> Agreeableness_w2 (ccc94_2)
161 Openness_w2 <> Agreeableness_w2 (ccc95_2)
162 Age <> Agreeableness_w2 (ccc96_2)
163 Agreeableness_w2 <> Unemployed_dummy (ccc97_2)
164 Agreeableness_w2 <> Retired_dummy (ccc98_2)
165 Female <> Agreeableness_w2 (ccc99_2)
166 Neuroticism_w2 <> Conscientiousness_w2 (ccc100_2)
167 Conscientiousness_w2 <> Openness_w2 (ccc101_2)
168 Conscientiousness_w2 <> Age (ccc102_2)
169 Conscientiousness_w2 <> Unemployed_dummy (ccc103_2)
170 Conscientiousness_w2 <> Retired_dummy (ccc104_2)
171 Conscientiousness_w2 <> Female (ccc105_2)
172 Neuroticism_w2 <> Openness_w2 (ccc106_2)
173 Neuroticism_w2 <> Age (ccc107_2)
174 Neuroticism_w2 <> Unemployed_dummy (ccc108_2)
175 Neuroticism_w2 <> Retired_dummy (ccc109_2)
176 Neuroticism_w2 <> Female (ccc110_2)
177 Openness_w2 <> Age (ccc111_2)
178 Openness_w2 <> Unemployed_dummy (ccc112_2)
179 Openness_w2 <> Retired_dummy (ccc113_2)
180 Openness_w2 <> Female (ccc114_2)
181 Age <> Unemployed_dummy (ccc115_2)
182 Age <> Retired_dummy (ccc116_2)
183 Age <> Female (ccc117_2)
184 Unemployed_dummy <> Retired_dummy (ccc118_2)
185 Female <> Unemployed_dummy (ccc119_2)
186 Female <> Retired_dummy (ccc120_2)
187 Loneliness_pl <> CorRestrOcc_w2 (ccc121_2)
188 CorRestrLeis_w2 <> Loneliness_pl (ccc122_2)
189 Loneliness_pl <> SM_Total_w1 (ccc123_2)
190 Loneliness_pl <> SM_Total_change (ccc124_2)
191 OtherMedia_Total_w1 <> Loneliness_pl (ccc125_2)
192 OtherMedia_Total_change <> Loneliness_pl (ccc126_2)
193 Education <> Loneliness_pl (ccc127_2)
194 Loneliness_pl <> Extraversion_w2 (ccc128_2)
195 Loneliness_pl <> Agreeableness_w2 (ccc129_2)
196 Conscientiousness_w2 <> Loneliness_pl (ccc130_2)

```
Neuroticism_w2 <> Loneliness_pl (ccc131_2)
Openness_w2 <> Loneliness_pl (ccc132_2)
Loneliness_pl <> Age (ccc133_2)
Loneliness_pl <> Unemployed_dummy (ccc134_2)
Loneliness_pl <> Retired_dummy (ccc135_2)
Loneliness_pl <> Female (ccc136_2)
Social_Media_Confidence_w2 <> Loneliness_pl (ccc137_2)
Social_Media_Confidence_w2 <> CorRestrOcc_w2 (ccc138_2)
CorRestrLeis_w2 <> Social_Media_Confidence_w2 (ccc139_2)
Social_Media_Confidence_w2 <> SM_Total_w1 (ccc140_2)
Social_Media_Confidence_w2 <> SM_Total_change (ccc141_2)
OtherMedia_Total_w1 <> Social_Media_Confidence_w2 (ccc142_2)
OtherMedia_Total_change <> Social_Media_Confidence_w2 (ccc143_2)
Education <> Social_Media_Confidence_w2 (ccc144_2)
Social_Media_Confidence_w2 <> Extraversion_w2 (ccc145_2)
Social_Media_Confidence_w2 <> Agreeableness_w2 (ccc146_2)
Conscientiousness_w2 <> Social_Media_Confidence_w2 (ccc147_2)
Neuroticism_w2 <> Social_Media_Confidence_w2 (ccc148_2)
Openness_w2 <> Social_Media_Confidence_w2 (ccc149_2)
Social_Media_Confidence_w2 <> Age (ccc150_2)
Social_Media_Confidence_w2 <> Unemployed_dummy (ccc151_2)
Social_Media_Confidence_w2 <> Retired_dummy (ccc152_2)
Social_Media_Confidence_w2 <> Female (ccc153_2)
NoFlatmates <> Social_Media_Confidence_w2 (ccc154_2)
NoFlatmates <> Loneliness_pl (ccc155_2)
NoFlatmates <> CorRestrOcc_w2 (ccc156_2)
CorRestrLeis_w2 <> NoFlatmates (ccc157_2)
NoFlatmates <> SM_Total_w1 (ccc158_2)
NoFlatmates <> SM_Total_change (ccc159_2)
OtherMedia_Total_w1 <> NoFlatmates (ccc160_2)
OtherMedia_Total_change <> NoFlatmates (ccc161_2)
Education <> NoFlatmates (ccc162_2)
NoFlatmates <> Extraversion_w2 (ccc163_2)
NoFlatmates <> Agreeableness_w2 (ccc164_2)
Conscientiousness_w2 <> NoFlatmates (ccc165_2)
Neuroticism_w2 <> NoFlatmates (ccc166_2)
Openness_w2 <> NoFlatmates (ccc167_2)
NoFlatmates <> Age (ccc168_2)
NoFlatmates <> Unemployed_dummy (ccc169_2)
NoFlatmates <> Retired_dummy (ccc170_2)
NoFlatmates <> Female (ccc171_2)
Commun_FacetoFace_change_c <> NoFlatmates (ccc172_2)
Commun_FacetoFace_change_c <> Social_Media_Confidence_w2 (ccc173_2)
Commun_FacetoFace_change_c <> Loneliness_pl (ccc174_2)
Commun_FacetoFace_change_c <> CorRestrOcc_w2 (ccc175_2)
CorRestrLeis_w2 <> Commun_FacetoFace_change_c (ccc176_2)
Commun_FacetoFace_change_c <> SM_Total_w1 (ccc177_2)
Commun_FacetoFace_change_c <> SM_Total_change (ccc178_2)
OtherMedia_Total_w1 <> Commun_FacetoFace_change_c (ccc179_2)
OtherMedia_Total_change <> Commun_FacetoFace_change_c (ccc180_2)
```

Education <> Commun_FacetoFace_change_c (ccc181_2)
Commun_FacetoFace_change_c <> Extraversion_w2 (ccc182_2)
Commun_FacetoFace_change_c <> Agreeableness_w2 (ccc183_2)
Conscientiousness_w2 <> Commun_FacetoFace_change_c (ccc184_2)
Neuroticism_w2 <> Commun_FacetoFace_change_c (ccc185_2)
Openness_w2 <> Commun_FacetoFace_change_c (ccc186_2)
Commun_FacetoFace_change_c <> Age (ccc187_2)
Commun_FacetoFace_change_c <> Unemployed_dummy (ccc188_2)
Commun_FacetoFace_change_c <> Retired_dummy (ccc189_2)
Commun_FacetoFace_change_c <> Female (ccc190_2)
Commun_VideoChats_change_c <> Commun_FacetoFace_change_c (ccc191_2)
Commun_VideoChats_change_c <> NoFlatmates (ccc192_2)
Commun_VideoChats_change_c <> Social_Media_Confidence_w2 (ccc193_2)
Commun_VideoChats_change_c <> Loneliness_pl (ccc194_2)
Commun_VideoChats_change_c <> CorRestrOcc_w2 (ccc195_2)
CorRestrLeis_w2 <> Commun_VideoChats_change_c (ccc196_2)
Commun_VideoChats_change_c <> SM_Total_w1 (ccc197_2)
Commun_VideoChats_change_c <> SM_Total_change (ccc198_2)
OtherMedia_Total_w1 <> Commun_VideoChats_change_c (ccc199_2)
OtherMedia_Total_change <> Commun_VideoChats_change_c (ccc200_2)
Education <> Commun_VideoChats_change_c (ccc201_2)
Commun_VideoChats_change_c <> Extraversion_w2 (ccc202_2)
Commun_VideoChats_change_c <> Agreeableness_w2 (ccc203_2)
Conscientiousness_w2 <> Commun_VideoChats_change_c (ccc204_2)
Neuroticism_w2 <> Commun_VideoChats_change_c (ccc205_2)
Openness_w2 <> Commun_VideoChats_change_c (ccc206_2)
Commun_VideoChats_change_c <> Age (ccc207_2)
Commun_VideoChats_change_c <> Unemployed_dummy (ccc208_2)
Commun_VideoChats_change_c <> Retired_dummy (ccc209_2)
Commun_VideoChats_change_c <> Female (ccc210_2)
Commun_VideoChats_change_c <> Commun_VideoChats_w0_c (ccc211_2)
Commun_VideoChats_w0_c <> Commun_FacetoFace_change_c (ccc212_2)
Commun_VideoChats_w0_c <> NoFlatmates (ccc213_2)
Commun_VideoChats_w0_c <> Social_Media_Confidence_w2 (ccc214_2)
Commun_VideoChats_w0_c <> Loneliness_pl (ccc215_2)
Commun_VideoChats_w0_c <> CorRestrOcc_w2 (ccc216_2)
CorRestrLeis_w2 <> Commun_VideoChats_w0_c (ccc217_2)
Commun_VideoChats_w0_c <> SM_Total_w1 (ccc218_2)
Commun_VideoChats_w0_c <> SM_Total_change (ccc219_2)
OtherMedia_Total_w1 <> Commun_VideoChats_w0_c (ccc220_2)
OtherMedia_Total_change <> Commun_VideoChats_w0_c (ccc221_2)
Education <> Commun_VideoChats_w0_c (ccc222_2)
Commun_VideoChats_w0_c <> Extraversion_w2 (ccc223_2)
Commun_VideoChats_w0_c <> Agreeableness_w2 (ccc224_2)
Conscientiousness_w2 <> Commun_VideoChats_w0_c (ccc225_2)
Neuroticism_w2 <> Commun_VideoChats_w0_c (ccc226_2)
Openness_w2 <> Commun_VideoChats_w0_c (ccc227_2)
Commun_VideoChats_w0_c <> Age (ccc228_2)
Commun_VideoChats_w0_c <> Unemployed_dummy (ccc229_2)
Commun_VideoChats_w0_c <> Retired_dummy (ccc230_2)

```
Commun_VideoChats_w0_c <> Female (ccc231_2)
Commun_FacetoFace_w0_c <> Commun_VideoChats_w0_c (ccc232_2)
Commun_VideoChats_change_c <> Commun_FacetoFace_w0_c (ccc233_2)
Commun_FacetoFace_w0_c <> Commun_FacetoFace_change_c (ccc234_2)
Commun_FacetoFace_w0_c <> NoFlatmates (ccc235_2)
Commun_FacetoFace_w0_c <> Social_Media_Confidence_w2 (ccc236_2)
Commun_FacetoFace_w0_c <> Loneliness_pl (ccc237_2)
Commun_FacetoFace_w0_c <> CorRestrOcc_w2 (ccc238_2)
CorRestrLeis_w2 <> Commun_FacetoFace_w0_c (ccc239_2)
Commun_FacetoFace_w0_c <> SM_Total_w1 (ccc240_2)
Commun_FacetoFace_w0_c <> SM_Total_change (ccc241_2)
OtherMedia_Total_w1 <> Commun_FacetoFace_w0_c (ccc242_2)
OtherMedia_Total_change <> Commun_FacetoFace_w0_c (ccc243_2)
Education <> Commun_FacetoFace_w0_c (ccc244_2)
Commun_FacetoFace_w0_c <> Extraversion_w2 (ccc245_2)
Commun_FacetoFace_w0_c <> Agreeableness_w2 (ccc246_2)
Conscientiousness_w2 <> Commun_FacetoFace_w0_c (ccc247_2)
Neuroticism_w2 <> Commun_FacetoFace_w0_c (ccc248_2)
Openness_w2 <> Commun_FacetoFace_w0_c (ccc249_2)
Commun_FacetoFace_w0_c <> Age (ccc250_2)
Commun_FacetoFace_w0_c <> Unemployed_dummy (ccc251_2)
Commun_FacetoFace_w0_c <> Retired_dummy (ccc252_2)
Commun_FacetoFace_w0_c <> Female (ccc253_2)
e2 <> e5 (c1_2)
e3 <> e6 (c2_2)
e4 <> e7 (c3_2)

Loneliness_pl (0), (vvv1_1)
e1 (0), (vv1_2)
e2 (0), (v1_2)
e3 (0), (v2_2)
e4 (0), (v3_2)
e5 (0), (v4_2)
e6 (0), (v5_2)
e7 (0), (v6_2)
SM_Total_change (), (vvv1_2)
SM_Total_w1 (), (vvv2_2)
Commun_FacetoFace_change_c (), (vvv3_2)
Commun_FacetoFace_w0_c (), (vvv4_2)
Commun_VideoChats_change_c (), (vvv5_2)
Commun_VideoChats_w0_c (), (vvv6_2)
OtherMedia_Total_change (), (vvv7_2)
OtherMedia_Total_w1 (), (vvv8_2)
CorRestrLeis_w2 (), (vvv9_2)
CorRestrOcc_w2 (), (vvv10_2)
Conscientiousness_w2 (), (vvv11_2)
Agreeableness_w2 (), (vvv12_2)
Extraversion_w2 (), (vvv13_2)
Neuroticism_w2 (), (vvv14_2)
Openness_w2 (), (vvv15_2)
```

```
Age (), (vvv16_2)
Female (), (vvv17_2)
Education (), (vvv18_2)
Unemployed_dummy (), (vvv19_2)
Retired_dummy (), (vvv20_2)
NoFlatmates (), (vvv21_2)
Social_Media_Confidence_w2 (), (vvv22_2)
```

S 2.3 Amos syntax post-hoc main model

```
Loneliness_I1_w1 = (int_1) + (1) e2 + (1) Loneliness_pl
Loneliness_I1_w2 = (int_2) + (1) e5 + (1) Loneliness_w2
Loneliness_I2_w1 = (int_3) + (w_2) Loneliness_pl + (1) e3
Loneliness_I2_w2 = (int_4) + (w_4) Loneliness_w2 + (1) e6
Loneliness_I3_w1 = (int_5) + (w_3) Loneliness_pl + (1) e4
Loneliness_I3_w2 = (int_6) + (1) e7 + (w_6) Loneliness_w2

Loneliness_pl (0), (vvv1_1)

Loneliness_w2 = (0) + SM_Total_change+ SM_Total_w1  +Commun_FacetoFace_change_c
+ Commun_FacetoFace_w0_c + Commun_VideoChats_change_c +
Commun_VideoChats_w0_c +   CorRestrLeis_w2 + CorRestrOcc_w2  +
OtherMedia_Total_change + OtherMedia_Total_w1+ Conscientiousness_w2 +
Agreeableness_w2 +Extraversion_w2 +  Neuroticism_w2 + Openness_w2  + Age+Female
+Education + Unemployed_dummy+ Retired_dummy + NoFlatmates+
Social_Media_Confidence_w2 + Loneliness_pl +
SM_Total_changexcommun_FacetoFace_change_MOD +
SM_Total_changexcommun_VideoChats_change_MOD   + (1) e1

CorRestrLeis_w2 <> CorRestrOcc_w2
CorRestrOcc_w2 <> SM_Total_w1
CorRestrOcc_w2 <> SM_Total_change
OtherMedia_Total_w1 <> CorRestrOcc_w2
CorRestrOcc_w2 <> OtherMedia_Total_change
Education <> CorRestrOcc_w2
Extraversion_w2 <> CorRestrOcc_w2
Agreeableness_w2 <> CorRestrOcc_w2
Conscientiousness_w2 <> CorRestrOcc_w2
Neuroticism_w2 <> CorRestrOcc_w2
Openness_w2 <> CorRestrOcc_w2
Age <> CorRestrOcc_w2
Unemployed_dummy <> CorRestrOcc_w2
Retired_dummy <> CorRestrOcc_w2
Female <> CorRestrOcc_w2
CorRestrLeis_w2 <> SM_Total_w1
CorRestrLeis_w2 <> SM_Total_change
OtherMedia_Total_w1 <> CorRestrLeis_w2
```

OtherMedia_Total_change <> CorRestrLeis_w2
Education <> CorRestrLeis_w2
CorRestrLeis_w2 <> Extraversion_w2
CorRestrLeis_w2 <> Agreeableness_w2
Conscientiousness_w2 <> CorRestrLeis_w2
Neuroticism_w2 <> CorRestrLeis_w2
Openness_w2 <> CorRestrLeis_w2
CorRestrLeis_w2 <> Age
CorRestrLeis_w2 <> Unemployed_dummy
CorRestrLeis_w2 <> Retired_dummy
CorRestrLeis_w2 <> Female
SM_Total_w1 <> SM_Total_change
SM_Total_w1 <> OtherMedia_Total_w1
SM_Total_w1 <> OtherMedia_Total_change
Education <> SM_Total_w1
Extraversion_w2 <> SM_Total_w1
Agreeableness_w2 <> SM_Total_w1
Conscientiousness_w2 <> SM_Total_w1
Neuroticism_w2 <> SM_Total_w1
Openness_w2 <> SM_Total_w1
Age <> SM_Total_w1
Unemployed_dummy <> SM_Total_w1
Retired_dummy <> SM_Total_w1
Female <> SM_Total_w1
SM_Total_change <> OtherMedia_Total_w1
SM_Total_change <> OtherMedia_Total_change
Education <> SM_Total_change
Extraversion_w2 <> SM_Total_change
Agreeableness_w2 <> SM_Total_change
Conscientiousness_w2 <> SM_Total_change
Neuroticism_w2 <> SM_Total_change
Openness_w2 <> SM_Total_change
Age <> SM_Total_change
Unemployed_dummy <> SM_Total_change
Retired_dummy <> SM_Total_change
Female <> SM_Total_change
OtherMedia_Total_change <> OtherMedia_Total_w1
OtherMedia_Total_w1 <> Education
OtherMedia_Total_w1 <> Extraversion_w2
OtherMedia_Total_w1 <> Agreeableness_w2
OtherMedia_Total_w1 <> Conscientiousness_w2
OtherMedia_Total_w1 <> Neuroticism_w2
OtherMedia_Total_w1 <> Openness_w2
OtherMedia_Total_w1 <> Age
OtherMedia_Total_w1 <> Unemployed_dummy
OtherMedia_Total_w1 <> Retired_dummy
OtherMedia_Total_w1 <> Female
OtherMedia_Total_change <> Education
OtherMedia_Total_change <> Extraversion_w2
OtherMedia_Total_change <> Agreeableness_w2

444 OtherMedia_Total_change <> Conscientiousness_w2
445 OtherMedia_Total_change <> Neuroticism_w2
446 OtherMedia_Total_change <> Openness_w2
447 OtherMedia_Total_change <> Age
448 OtherMedia_Total_change <> Unemployed_dummy
449 OtherMedia_Total_change <> Retired_dummy
450 OtherMedia_Total_change <> Female
451 Education <> Extraversion_w2
452 Education <> Agreeableness_w2
453 Education <> Conscientiousness_w2
454 Education <> Neuroticism_w2
455 Education <> Openness_w2
456 Education <> Age
457 Education <> Unemployed_dummy
458 Education <> Retired_dummy
459 Education <> Female
460 Agreeableness_w2 <> Extraversion_w2
461 Conscientiousness_w2 <> Extraversion_w2
462 Neuroticism_w2 <> Extraversion_w2
463 Openness_w2 <> Extraversion_w2
464 Age <> Extraversion_w2
465 Unemployed_dummy <> Extraversion_w2
466 Retired_dummy <> Extraversion_w2
467 Female <> Extraversion_w2
468 Conscientiousness_w2 <> Agreeableness_w2
469 Neuroticism_w2 <> Agreeableness_w2
470 Openness_w2 <> Agreeableness_w2
471 Age <> Agreeableness_w2
472 Agreeableness_w2 <> Unemployed_dummy
473 Agreeableness_w2 <> Retired_dummy
474 Female <> Agreeableness_w2
475 Neuroticism_w2 <> Conscientiousness_w2
476 Conscientiousness_w2 <> Openness_w2
477 Conscientiousness_w2 <> Age
478 Conscientiousness_w2 <> Unemployed_dummy
479 Conscientiousness_w2 <> Retired_dummy
480 Conscientiousness_w2 <> Female
481 Neuroticism_w2 <> Openness_w2
482 Neuroticism_w2 <> Age
483 Neuroticism_w2 <> Unemployed_dummy
484 Neuroticism_w2 <> Retired_dummy
485 Neuroticism_w2 <> Female
486 Openness_w2 <> Age
487 Openness_w2 <> Unemployed_dummy
488 Openness_w2 <> Retired_dummy
489 Openness_w2 <> Female
490 Age <> Unemployed_dummy
491 Age <> Retired_dummy
492 Age <> Female
493 Unemployed_dummy <> Retired_dummy

Female <> Unemployed_dummy
Female <> Retired_dummy
Loneliness_pl <> CorRestrOcc_w2
CorRestrLeis_w2 <> Loneliness_pl
Loneliness_pl <> SM_Total_w1
Loneliness_pl <> SM_Total_change
OtherMedia_Total_w1 <> Loneliness_pl
OtherMedia_Total_change <> Loneliness_pl
Education <> Loneliness_pl
Loneliness_pl <> Extraversion_w2
Loneliness_pl <> Agreeableness_w2
Conscientiousness_w2 <> Loneliness_pl
Neuroticism_w2 <> Loneliness_pl
Openness_w2 <> Loneliness_pl
Loneliness_pl <> Age
Loneliness_pl <> Unemployed_dummy
Loneliness_pl <> Retired_dummy
Loneliness_pl <> Female
Social_Media_Confidence_w2 <> Loneliness_pl
Social_Media_Confidence_w2 <> CorRestrOcc_w2
CorRestrLeis_w2 <> Social_Media_Confidence_w2
Social_Media_Confidence_w2 <> SM_Total_w1
Social_Media_Confidence_w2 <> SM_Total_change
OtherMedia_Total_w1 <> Social_Media_Confidence_w2
OtherMedia_Total_change <> Social_Media_Confidence_w2
Education <> Social_Media_Confidence_w2
Social_Media_Confidence_w2 <> Extraversion_w2
Social_Media_Confidence_w2 <> Agreeableness_w2
Conscientiousness_w2 <> Social_Media_Confidence_w2
Neuroticism_w2 <> Social_Media_Confidence_w2
Openness_w2 <> Social_Media_Confidence_w2
Social_Media_Confidence_w2 <> Age
Social_Media_Confidence_w2 <> Unemployed_dummy
Social_Media_Confidence_w2 <> Retired_dummy
Social_Media_Confidence_w2 <> Female
NoFlatmates <> Social_Media_Confidence_w2
NoFlatmates <> Loneliness_pl
NoFlatmates <> CorRestrOcc_w2
CorRestrLeis_w2 <> NoFlatmates
NoFlatmates <> SM_Total_w1
NoFlatmates <> SM_Total_change
OtherMedia_Total_w1 <> NoFlatmates
OtherMedia_Total_change <> NoFlatmates
Education <> NoFlatmates
NoFlatmates <> Extraversion_w2
NoFlatmates <> Agreeableness_w2
Conscientiousness_w2 <> NoFlatmates
Neuroticism_w2 <> NoFlatmates
Openness_w2 <> NoFlatmates
NoFlatmates <> Age

```
NoFlatmates <> Unemployed_dummy
NoFlatmates <> Retired_dummy
NoFlatmates <> Female
SM_Total_changexcommun_VideoChats_change_MOD <> NoFlatmates
SM_Total_changexcommun_VideoChats_change_MOD <> Social_Media_Confidence_w2
SM_Total_changexcommun_VideoChats_change_MOD <> Loneliness_pl
SM_Total_changexcommun_VideoChats_change_MOD <> CorRestrOcc_w2
CorRestrLeis_w2 <> SM_Total_changexcommun_VideoChats_change_MOD
SM_Total_changexcommun_VideoChats_change_MOD <> SM_Total_w1
SM_Total_changexcommun_VideoChats_change_MOD <> SM_Total_change
OtherMedia_Total_w1 <> SM_Total_changexcommun_VideoChats_change_MOD
OtherMedia_Total_change <> SM_Total_changexcommun_VideoChats_change_MOD
Education <> SM_Total_changexcommun_VideoChats_change_MOD
SM_Total_changexcommun_VideoChats_change_MOD <> Extraversion_w2
SM_Total_changexcommun_VideoChats_change_MOD <> Agreeableness_w2
Conscientiousness_w2 <> SM_Total_changexcommun_VideoChats_change_MOD
Neuroticism_w2 <> SM_Total_changexcommun_VideoChats_change_MOD
Openness_w2 <> SM_Total_changexcommun_VideoChats_change_MOD
SM_Total_changexcommun_VideoChats_change_MOD <> Age
SM_Total_changexcommun_VideoChats_change_MOD <> Unemployed_dummy
SM_Total_changexcommun_VideoChats_change_MOD <> Retired_dummy
SM_Total_changexcommun_VideoChats_change_MOD <> Female
Commun_FacetoFace_w0_c <> SM_Total_changexcommun_VideoChats_change_MOD
Commun_FacetoFace_w0_c <> NoFlatmates
Commun_FacetoFace_w0_c <> Social_Media_Confidence_w2
Commun_FacetoFace_w0_c <> Loneliness_pl
Commun_FacetoFace_w0_c <> CorRestrOcc_w2
CorRestrLeis_w2 <> Commun_FacetoFace_w0_c
Commun_FacetoFace_w0_c <> SM_Total_w1
Commun_FacetoFace_w0_c <> SM_Total_change
OtherMedia_Total_w1 <> Commun_FacetoFace_w0_c
OtherMedia_Total_change <> Commun_FacetoFace_w0_c
Education <> Commun_FacetoFace_w0_c
Commun_FacetoFace_w0_c <> Extraversion_w2
Commun_FacetoFace_w0_c <> Agreeableness_w2
Conscientiousness_w2 <> Commun_FacetoFace_w0_c
Neuroticism_w2 <> Commun_FacetoFace_w0_c
Openness_w2 <> Commun_FacetoFace_w0_c
Commun_FacetoFace_w0_c <> Age
Commun_FacetoFace_w0_c <> Unemployed_dummy
Commun_FacetoFace_w0_c <> Retired_dummy
Commun_FacetoFace_w0_c <> Female
Commun_VideoChats_w0_c <> Commun_FacetoFace_w0_c
Commun_VideoChats_w0_c <> SM_Total_changexcommun_VideoChats_change_MOD
Commun_VideoChats_w0_c <> NoFlatmates
Commun_VideoChats_w0_c <> Social_Media_Confidence_w2
Commun_VideoChats_w0_c <> Loneliness_pl
Commun_VideoChats_w0_c <> CorRestrOcc_w2
CorRestrLeis_w2 <> Commun_VideoChats_w0_c
Commun_VideoChats_w0_c <> SM_Total_w1
```

Commun_VideoChats_w0_c <> SM_Total_change
OtherMedia_Total_w1 <> Commun_VideoChats_w0_c
OtherMedia_Total_change <> Commun_VideoChats_w0_c
Education <> Commun_VideoChats_w0_c
Commun_VideoChats_w0_c <> Extraversion_w2
Commun_VideoChats_w0_c <> Agreeableness_w2
Conscientiousness_w2 <> Commun_VideoChats_w0_c
Neuroticism_w2 <> Commun_VideoChats_w0_c
Openness_w2 <> Commun_VideoChats_w0_c
Commun_VideoChats_w0_c <> Age
Commun_VideoChats_w0_c <> Unemployed_dummy
Commun_VideoChats_w0_c <> Retired_dummy
Commun_VideoChats_w0_c <> Female
Commun_VideoChats_change_c <> SM_Total_changexcommun_VideoChats_change_MOD
Commun_VideoChats_change_c <> NoFlatmates
Commun_VideoChats_change_c <> Social_Media_Confidence_w2
Commun_VideoChats_change_c <> Loneliness_pl
Commun_VideoChats_change_c <> CorRestrOcc_w2
CorRestrLeis_w2 <> Commun_VideoChats_change_c
Commun_VideoChats_change_c <> SM_Total_w1
Commun_VideoChats_change_c <> SM_Total_change
OtherMedia_Total_w1 <> Commun_VideoChats_change_c
OtherMedia_Total_change <> Commun_VideoChats_change_c
Education <> Commun_VideoChats_change_c
Commun_VideoChats_change_c <> Extraversion_w2
Commun_VideoChats_change_c <> Agreeableness_w2
Conscientiousness_w2 <> Commun_VideoChats_change_c
Neuroticism_w2 <> Commun_VideoChats_change_c
Openness_w2 <> Commun_VideoChats_change_c
Commun_VideoChats_change_c <> Age
Commun_VideoChats_change_c <> Unemployed_dummy
Commun_VideoChats_change_c <> Retired_dummy
Commun_VideoChats_change_c <> Female
Commun_VideoChats_change_c <> Commun_FacetoFace_w0_c
Commun_VideoChats_change_c <> Commun_VideoChats_w0_c
Commun_FacetoFace_change_c <> Commun_VideoChats_change_c
Commun_FacetoFace_change_c <> SM_Total_changexcommun_VideoChats_change_MOD
Commun_FacetoFace_change_c <> NoFlatmates
Commun_FacetoFace_change_c <> Social_Media_Confidence_w2
Commun_FacetoFace_change_c <> Loneliness_pl
Commun_FacetoFace_change_c <> CorRestrOcc_w2
CorRestrLeis_w2 <> Commun_FacetoFace_change_c
Commun_FacetoFace_change_c <> SM_Total_w1
Commun_FacetoFace_change_c <> SM_Total_change
OtherMedia_Total_w1 <> Commun_FacetoFace_change_c
OtherMedia_Total_change <> Commun_FacetoFace_change_c
Education <> Commun_FacetoFace_change_c
Commun_FacetoFace_change_c <> Extraversion_w2
Commun_FacetoFace_change_c <> Agreeableness_w2

Conscientiousness_w2 <> Commun_FacetoFace_change_c
Neuroticism_w2 <> Commun_FacetoFace_change_c
Openness_w2 <> Commun_FacetoFace_change_c
Commun_FacetoFace_change_c <> Age
Commun_FacetoFace_change_c <> Unemployed_dummy
Commun_FacetoFace_change_c <> Retired_dummy
Commun_FacetoFace_change_c <> Female
Commun_FacetoFace_change_c <> Commun_FacetoFace_w0_c
Commun_FacetoFace_change_c <> Commun_VideoChats_w0_c
SM_Total_changexcommun_FacetoFace_change_MOD <>
SM_Total_changexcommun_VideoChats_change_MOD
SM_Total_changexcommun_FacetoFace_change_MOD <> Commun_VideoChats_w0_c
SM_Total_changexcommun_FacetoFace_change_MOD <> Commun_FacetoFace_w0_c
SM_Total_changexcommun_FacetoFace_change_MOD <> Commun_VideoChats_change_c
SM_Total_changexcommun_FacetoFace_change_MOD <> Commun_FacetoFace_change_c
SM_Total_changexcommun_FacetoFace_change_MOD <> NoFlatmates
SM_Total_changexcommun_FacetoFace_change_MOD <> Social_Media_Confidence_w2
SM_Total_changexcommun_FacetoFace_change_MOD <> Loneliness_pl
SM_Total_changexcommun_FacetoFace_change_MOD <> CorRestrOcc_w2
CorRestrLeis_w2 <> SM_Total_changexcommun_FacetoFace_change_MOD
SM_Total_changexcommun_FacetoFace_change_MOD <> SM_Total_w1
SM_Total_changexcommun_FacetoFace_change_MOD <> SM_Total_change
OtherMedia_Total_w1 <> SM_Total_changexcommun_FacetoFace_change_MOD
OtherMedia_Total_change <> SM_Total_changexcommun_FacetoFace_change_MOD
Education <> SM_Total_changexcommun_FacetoFace_change_MOD
SM_Total_changexcommun_FacetoFace_change_MOD <> Extraversion_w2
SM_Total_changexcommun_FacetoFace_change_MOD <> Agreeableness_w2
Conscientiousness_w2 <> SM_Total_changexcommun_FacetoFace_change_MOD
Neuroticism_w2 <> SM_Total_changexcommun_FacetoFace_change_MOD
Openness_w2 <> SM_Total_changexcommun_FacetoFace_change_MOD
SM_Total_changexcommun_FacetoFace_change_MOD <> Age
SM_Total_changexcommun_FacetoFace_change_MOD <> Unemployed_dummy
SM_Total_changexcommun_FacetoFace_change_MOD <> Retired_dummy
SM_Total_changexcommun_FacetoFace_change_MOD <> Female
e2 <> e5 (c1_2)
e3 <> e6 (c2_2)
e4 <> e7 (c3_2)

e1 (0), (vv1_2)
e2 (0), (v1_2)
e3 (0), (v2_2)
e4 (0), (v3_2)
e5 (0), (v4_2)
e6 (0), (v5_2)
e7 (0), (v6_2)
